# Breeding for colored quality protein popcorn with improved amino acid composition

**DOI:** 10.3389/fpls.2026.1844370

**Published:** 2026-05-25

**Authors:** Jonathan Niyorukundo, Abou Yobi, Cephas Sithole, Caleb Wehrbein, Ruthie Angelovici, David R. Holding

**Affiliations:** 1 Department of Agronomy and Horticulture, University of Nebraska, Lincoln, NE, United States; 2 Department of Biological Sciences, University of Missouri, Columbia, MO, United States

**Keywords:** lysine, maize kernel color, popcorn, proteome rebalancing, quality protein maize, quality protein popcorn, zeins

## Abstract

Popcorn is deficient in essential lysine due to the high abundance of lysine-devoid zein storage proteins in the endosperm. *Opaque-2* (*o2*) mutants reduce zein accumulation, resulting in increased lysine-containing non-zein proteins. By introgressing *o2* along with modifier genes into dent corn and popcorn germplasm, high-lysine varieties called Quality Protein Maize (QPM) and Quality Protein Popcorn (QPP) have been created. While previous QPP studies used proprietary yellow popcorn germplasm, we employed publicly available, highly open-pollinated, and diversely colored popcorn germplasm. The objective of this project was to breed high-lysine, diversely colored popcorn inbreds with improved protein quality, which can then be used to produce QPP hybrids with diverse flake taste, aroma, and texture, along with robust agronomics. QPM lines were crossed with colored popcorns to generate novel colored QPP lines. Segregating F_2_ ears were used for selection of diverse color, popcorn kernel size and shape, and *o2* phenotypes. Using recurrent backcrossing and selection to the BC_3_ generation, followed by five generations of selfing and selection, nine BC_3_F_5_ colored QPP inbreds were selected for analysis and hybrid crossing. SDS-PAGE analysis confirmed the reduction of the 22-kDa alpha zeins, and despite the low alpha zeins, protein concentrations remained equivalent to the parental popcorns. Analysis of the amino acids revealed a significant increase in protein-bound lysine (up to twofold), free lysine (up to 10-fold), and variable increases in free tryptophan in popped QPP inbreds. The inbreds exhibited variability in popping performance, with five of the nine exhibiting significant reductions in pop volume, while four inbreds produced popping values similar to parental popcorn lines and were selected for ongoing production of QPP hybrids. This study highlights the potential to address popcorn’s incomplete protein profile by increasing essential lysine content, thereby expanding its nutritional potential. Superior colored QPP genotypes bearing both normal popcorn endosperm vitreousness and high-lysine trait characteristics are currently being used in the development of various colored QPP hybrids.

## Introduction

Popcorn (Zea mays everta) is a low-calorie, high-fiber whole-grain snack consumed exclusively by humans worldwide. In the United States, it accounts for 17% of total whole-grain intake (Cervantes-López et al., 2025). Popcorn differs from other types of maize due to its highly vitreous endosperm and hard pericarp (Scott et al., 2019). Similar to other maize types, endosperm storage proteins in popcorn are composed of approximately 60% zein storage proteins (Li and Song, 2020). Zein storage proteins are synthesized in the endoplasmic reticulum (ER) and are classified based on their molecular weight: alpha zeins (19 and 22 kDa), beta zeins (15 kDa), gamma zeins (16, 27, and 50 kDa), and delta zeins (10 and 18 kDa) (Schmidt et al., 1992; Reyes et al., 2011). The alpha zeins, which make up 70% of total zeins (Gibbon et al., 2003; Wu and Messing, 2010; Li and Song, 2020), are mainly regulated by the OPAQUE-2 transcription factor, which primarily regulates the synthesis of the 22 kDa fraction of alpha zeins (Kodrzycki et al., 1989; Schmidt et al., 1990; Yue et al., 2014). The alpha zeins are deficient in the limiting essential amino acids lysine and tryptophan, leading to an incomplete grain protein profile. Seed lysine content is one of the primary factors determining nutritional quality in maize (Babu et al., 2015). Zein content, lysine-containing non-zeins, and free lysine determine total lysine content (Habben et al., 1993). Improving popcorn’s incomplete protein profile would elevate its overall nutritional value and potentially increase its market potential in the expanding healthy snack sector.

In the 1960s, the maize opaque-2 (o2) mutation was reported to reduce the synthesis of the 22 kDa alpha zeins while increasing other non-zein proteins (Kodrzycki et al., 1989). Opaque-2 mutants were shown to have doubled lysine and tryptophan content (Mertz et al., 1964). However, o2 posed adverse pleiotropic effects, such as soft, opaque kernel endosperm, which conferred susceptibility to insect damage and seed breakage (Ortega and Bates, 1983). Researchers at CIMMYT, Mexico, selected for unknown o2 modifiers to recover hard kernel endosperm and developed high-lysine, hard-endosperm cultivars known as Quality Protein Maize (QPM) (Villegas et al., 1992). Several mapping studies identified Quantitative Trait Locus (QTLs) for modifiers (in terms of kernel vitreousness and density) on chromosomes 1, 5, 7, and 9 (Babu et al., 2005; Holding et al., 2008, 2011). An increase in gene expression and protein accumulation of 27-kDa γ-zein plays a dominant role in modification in QPM, and this gene forms the basis for the major QTL on Chromosome 7 (Geetha et al., 1991; Holding, 2014; Holding and Larkins, 2008; Wu and Messing, 2010). The increase in 27-kDa γ-zein has been shown to result from gene duplication and is present in all QPMs and many wild type popcorn varieties (Liu et al., 2016). The 27-kDa γ-zein increase is thus a convenient and reliable biochemical marker for endosperm modification in QPM.

Decades after the development of QPM, o2 and modifier genes were introgressed into elite popcorn germplasm, leading to the successful development of Quality Protein Popcorn (QPP) inbreds (Ren et al., 2018). These inbreds were further evaluated, selected, and hybridized to produce competitively performing but proprietary QPP hybrids (Parsons et al., 2020, 2021a). Consumer testing of novel QPP lines showed variation in taste, texture, and overall likability of QPP hybrids compared with commercial non-QPP hybrids (Parsons et al., 2021b). Serendipitously, in vitro fermentation of the popped QPP lines showed improved human gut microbiome activity (Korth et al., 2022).

Proprietary QPP lines (Ren et al., 2018; Parsons et al., 2020, 2021a) and colored popcorns, such as red, blue, yellow, white, brown, green, and purple (Lago et al., 2013; Bayomy, 2017), exist. However, colored QPP lines have not been reported. Because commercial popcorn varieties are primarily yellow and white (Scott et al., 2019), we sought to introgress the o2 mutation into non-proprietary colored popcorn germplasm to further diversify flake taste, texture, and nutritional quality profiles, while also further exploring microbiome effects and while the added nutritional potential of kernel pigmentation. Pigmented maize accumulates kernel phenolic, flavonoid, and carotenoid compounds. These compounds have been linked to various health-promoting benefits due to their highly antioxidant, anti-inflammatory, and anti-carcinogenic properties, which have been shown to reduce age-related macular degeneration, cataracts, cancer risk, diabetes, and cardiovascular diseases (Johnson, 2012; Lago et al., 2013; Milani et al., 2017; Eggersdorfer and Wyss, 2018). Kernel pigments, combined with high lysine, position colored QPP as a functional food. The Food and Agriculture Organization (FAO) of the United Nations (UN) describes functional food as those that provide additional health-promoting nutrients beyond the basic nutrition (FAO, 2022). Popcorn is often sold with abundant additives, including artificial colorants, saturated fats, salt, and sugar, to appeal to various consumer preferences. However, these additives diminish the inherent healthfulness for which popcorn is praised. With the benefits linked to maize pigments and the inherent improved taste, texture, and gut microbiome activity associated with QPP (Parsons et al, 2021b; Korth et al., 2022), colored QPP lines have strong potential through enhanced nutritional value, visual appeal, and potentially increased market demand in the healthy snack market.

In this study, our objective was to breed the high-lysine trait into colored popcorn germplasm to generate novel colored QPP inbreds. Furthermore, by using a diverse array of public germplasm, the aim was to generate new inbreds from non-proprietary parents that can later be used to generate hybrids with even greater taste and nutritional diversity. We initiated production of colored QPP inbreds using a conventional breeding approach in which two QPM lines (o2 donors) were crossed with six colored popcorn lines. At each generation, segregating QPP populations were selected for kernel color phenotypes, the o2 mutation, and vitreous endosperm modifiers. The resultant BC3F5 colored QPP inbreds were analyzed and compared with normal colored popcorns for changes in amino acids profile, total protein concentration, kernel endosperm modification, and popping volume. Results indicated that lysine increased significantly in colored QPP inbreds relative to normal popcorns. Additionally, total protein concentrations were not affected. However, a significant reduction in average popping quality was observed in five of the nine colored QPP inbreds. The resulting colored QPP inbreds, bearing desired kernel color, vitreousness and good popping flake volume, were selected as favor candidates for ongoing development of new colored high-quality protein popcorn hybrids.

## Materials and methods

### Planting, site location, and field design

Colored popcorn and QPM parent lines were obtained from the public seed stock center and small seed suppliers (Mary’s Heirloom Seeds, Sherwood’s Seeds, and Baker Creek Heirloom Seeds). Two QPM and six colored popcorn lines were acquired. The QPM parents were CML154 and TX807, and the colored popcorn lines (P1-P6) were Red popcorn, Cherokee Long Ear Popcorn (CLEP), Cochiti Pueblo Popcorn (CPP), Black Jewel, Glass Gem, and Mini Blue Popcorn, respectively. The planting site had a Kennebec silty clay soil type and was located in a temperate climate zone at the University of Nebraska-Lincoln East Campus field in Lincoln, NE (40.83486°N, 96.66377°W). The field was rain-irrigated during the summers of 2020 and 2021. Drip tape irrigation was used during the summers of 2022-2024. QPP introgressions were planted in a single row 4.5 m long, spaced 76cm apart. A minimum of 20 plants per genotype were planted for implicit replication during the early generations of selfing and backcrossing. Fields were treated with emergent herbicide (Acuron at 2.5qt per acre), and 150 lb of nitrogen was applied as dry urea before planting. The field was hand-hoed post-planting to manage weeds. A maize-soybean crop rotation was used to manage pest and weed pressure.

### QPP breeding scheme

**Figure 1:**
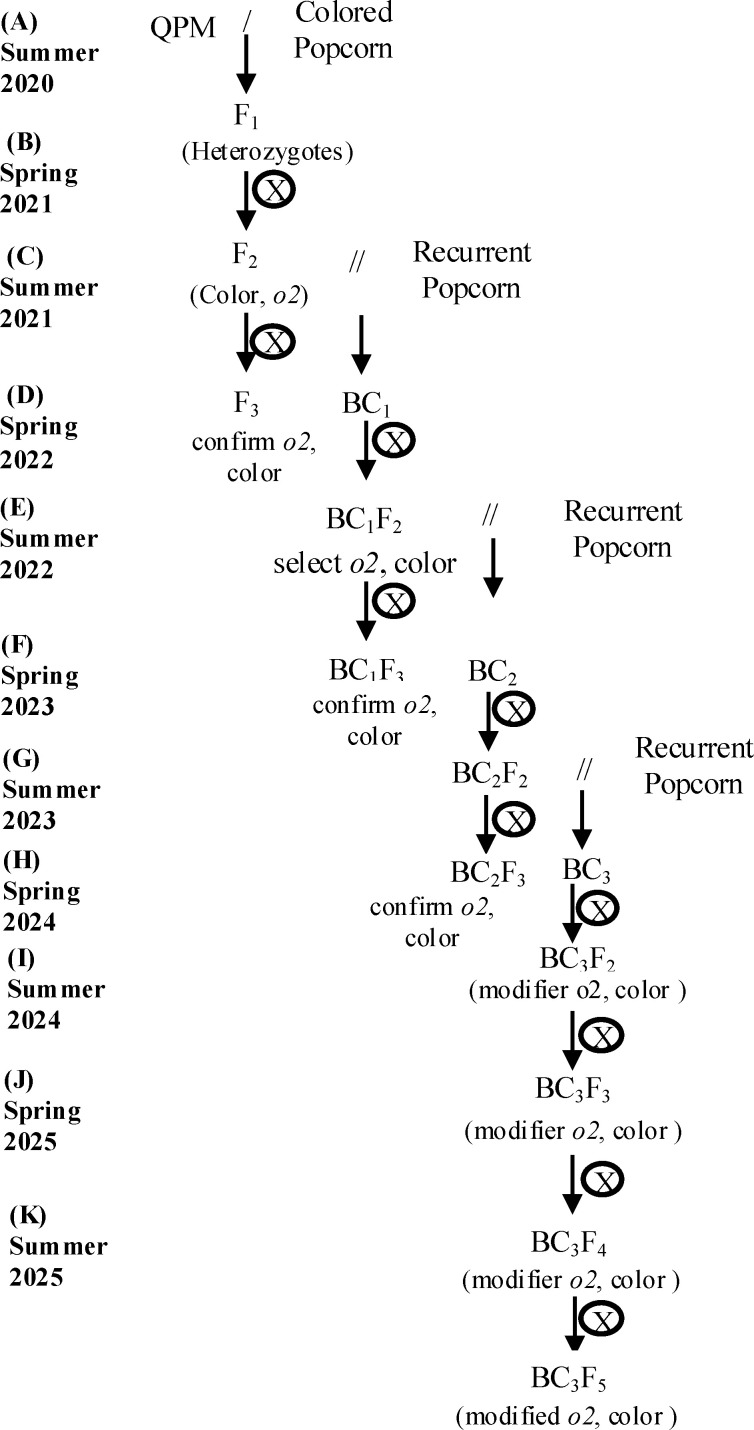
Colored QPP breeding scheme. Panels **(A-K)** represent the steps in the breeding scheme. **(A)** Unidirectional cross between QPM (female) and popcorn (male). **(B)** Self-pollinated F1 lines. **(C)** Segregation of color and o2 phenotypes in the F2 population; plants were confirmed and selected o2 F2 using SDS-PAGE, then self-pollinated and backcrossed to recurrent popcorn. **(D)** confirm o2 and color phenotype in the F3 population and self-pollinate the corresponding BC1. **(E)** confirm and select colored o2 BC1F2 and simultaneously self-pollinate and backcross to the recurrent popcorn. **(F)** confirm o2 and color diversity in BC1F3 and self-pollinate BC2. **(G)** confirm and select colored o2 BC2F2 and self-pollinate and backcross to recurrent popcorn. **(H)** confirm o2 in BC2F3 and self-pollinate BC3. **(I)** confirm and select color and o2 then self-pollinate to make BC3F3. **(J)** confirm and select color and o2 then self-pollinate to make BC3F4. **(K)** confirm and select color and o2 then self-pollinate to make BC3F5.

The breeding scheme for the introgression of colored popcorn and QPM parents used in the development of colored QPP inbreds is shown in **Figure 1**. In the summer of 2020, initial crosses were performed between two QPM and six colored popcorn parents (**Table 1**). Popcorn and QPM parents were crossed unidirectionally in the field as males and females, respectively, due to popcorn’s Ga1-s trait (Moran Lauter et al., 2022). From 2021 to 2023, starting with the F2 segregating populations, a minimum of 12 different plants per QPP introgression were self-pollinated in the greenhouse during the winter, followed by simultaneous self-pollination and backcrossing of at least 20 plants per QPP introgression to the recurrent popcorn parent in the field during the summer. Three consecutive generations were used to produce BC3 colored QPP populations. The fixation of o2, opm, and kernel pigments was carried out in the greenhouse during the winter and in the field during the summer from 2024 to 2025. BC3 populations were self-pollinated for five consecutive generations to produce the final BC3F5 QPP inbreds (**Table 2**).

**Table 1:** List of F_1_ QPP parental crosses.

Parent Crosses	P1 red popcorn	P2 cherokee long ear popcorn	P3 cochiti pueblo popcorn	P4 black jewel popcorn	P5 glass gem popcorn	P6 mini blue popcorn
QPM1 CML154	4	9	3	8	21	–
QPM2 TX807	1	–	–	3	8	–

Rows marked with (-) indicates an unknown number of parental crosses.

**Table 2:** Breeding pedigree of the QPP inbred lines.

QPP inbreds	Pedigree
QPP1	(QPM1/P1) → F1 → F2 → (F2//P1) → BC1 → BC1F2 → (BC1F2//P1) → BC2→ BC2F2 → (BC2F2//P1) → BC3 → BC3F5
QPP2	(QPM1/P2) → F1 → F2 → (F2//P2) → BC1 → BC1F2 → (BC1F2//P2) → BC2→ BC2F2 → (BC2F2//P2) → BC3 → BC3F5
QPP3	(QPM1/P3) → F1 → F2 → (F2//P3) → BC1 → BC1F2 → (BC1F2//P3) → BC2→ BC2F2 → (BC2F2//P3) → BC3 → BC3F5
QPP4	(QPM1/P4) → F1 → F2 → (F2//P4) → BC1 → BC1F2 → (BC1F2//P4) → BC2→ BC2F2 → (BC2F2//P4) → BC3 → BC3F5
QPP5	(QPM2/P1) → F1 → F2 → (F2//P1) → BC1 → BC1F2 → (BC1F2//P1) → BC2→ BC2F2 → (BC2F2//P1) → BC3 → BC3F5
QPP6	(QPM2/P2) → F1 → F2 → (F2//P2) → BC1 → BC1F2 → (BC1F2//P2) → BC2→ BC2F2 → (BC2F2//P2) → BC3 → BC3F5
QPP7	(QPM2/P4) → F1 → F2 → (F2//P4) → BC1 → BC1F2 → (BC1F2//P4) → BC2→ BC2F2 → (BC2F2//P4) → BC3 → BC3F5
QPP8	(QPM2/P5) → F1 → F2 → (F2//P5) → BC1 → BC1F2 → (BC1F2//P5) → BC2→ BC2F2 → (BC2F2//P5) → BC3 → BC3F5
QPP9	(QPM2/P6) → F1 → F2 → (F2//P6) → BC1 → BC1F2 → (BC1F2//P6) → BC2→ BC2F5

QPM, Quality Protein Maize, o2 donor; P, parental popcorn. QPM/P crosses initiated the development of QPP inbreds.

### Selection of kernel color and *opaque-2* modifier phenotypes

**Figure 2:**
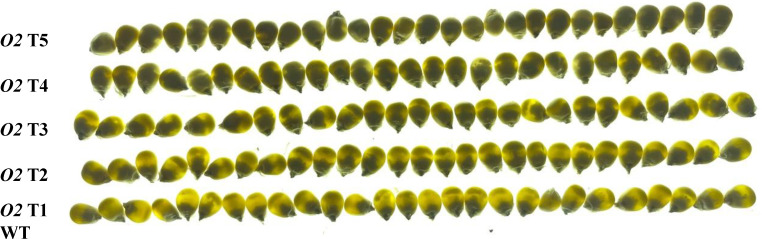
Lightbox screening for opaque endosperm modifiers in colored QPP inbreds.

Pigmented QPPs were selected for diverse kernel colors, such as red, purple, black, and blue. Yellow and white QPPs were also included in the selection and served as non-colored controls (**Supplementary Figures 1**–**4**). The selection of opm phenotypes and levels of endosperm modification were visually identified and selected in yellow and white kernels with the use of a lightbox (**Figure 2**). The o2 genotype forms a unique white spot visible on the kernel pericarp and can be easily identified visually in yellow and white kernels (**Supplementary Figure 7**). In colored QPP introgressions, the color phenotype obscured the o2 kernel phenotype; therefore, a row of kernels was ground to expose the internal endosperm and visualize the o2 endosperm modification (**Supplementary Figure 8**). The endosperm modification Type 2 was selected to ensure optimal kernel popping.

### Zein extraction and SDS PAGE confirmation of *opaque-2*

Previously used PCR markers for o2, such as UMC1066 (Babu et al., 2005), were not reliable for distinguishing between WT and mutant alleles for the o2 donors used in this study. However, very low accumulation of alpha zeins compared with wild-type popcorn is a quick and reliable marker for o2. Zein proteins were profiled using SDS-PAGE analysis to confirm o2 in the selected QPP introgressions. Zeins were extracted as described and modified by Wallace et al. (1990). Three kernels per QPP genotype were randomly selected from chosen ears and ground into fine flour samples. A total of 50 mg QPP flour samples were mixed with 1.6 mL borate buffer (12.5 mM Sodium Borate, 1% SDS at pH 10) containing 2% β-mercaptoethanol and incubated at room temperature (20-25°C) with shaking (40–60 rpm) for 1–2 h. Samples were centrifuged for 15 min at 13,300 rpm. The supernatant (total protein extract) was transferred to a new tube. Then, 700 μL ethanol was added to 300 μL total protein extract (70% final ethanol concentration) to precipitate non-zeins. Samples were incubated in the fridge at 4°C overnight. After centrifugation for 15 min at room temperature (20-25°C), the supernatant (zein extract) was transferred to a new tube. Next, 100 μL zein extract was dried for 1–2 h in a SpeedVac and resuspended in 40 μL 1x SDS loading buffer. Finally, 5 μL samples were used in SDS-PAGE analysis to profile the zeins.

### Total protein quantification

Total proteins (zeins and non-zeins) were extracted at the pre-ethanol precipitation step of the aforementioned zein extraction protocol. Five biological replicates per QPP genotype were used. Three kernels were randomly pooled from each replicate. Total protein concentrations were measured using the bicinchoninic acid (BCA) protein assay. Bovine serum albumin (BSA) standards and working reagents were provided with the BCA assay kit. QPP samples were diluted 100-fold to ensure the concentrations fell within the standard curve range of 0-2000 μg/mL and were used for BCA analysis following the manufacturer’s protocol. Briefly, 100 μL of diluted sample and nine BSA standards of concentrations ranging from 0-2000 μg/mL were added to 2 mL of BCA working reagent (50:1, reagent A: B). The solutions were incubated at room temperature (15-30°C) for 2 h and covered to prevent light exposure. Sample and standards absorbances were immediately measured at 652 nm using the BIO-RAD SmartSpec™ Plus spectrophotometer. Sample absorbances were read against the standard curve to measure protein concentrations of QPP inbreds, QPM, and popcorn parents.

### Amino acid profiling

Protein-bound amino acids (PBAA) and free amino acids (FAA) were extracted and analyzed as previously described (Angelovici et al., 2013; Ansaf et al., 2025a, 2025b). For non-popped kernels, three dry kernels were pooled from five biological replicates per genotype. Samples were collected from two QPM, six popcorn, and nine QPP inbred lines and ground into fine flour. For popped samples, the same pooling strategy was used, but kernels were first air popped before being ground into fine flour using a mortar and pestle with liquid nitrogen. Subsequently, 3 mg and 4 mg samples were used for extraction of PBAA and FAA, respectively. For FAA extraction, samples were extracted with Milli-Q purified water containing 13 internal standards. Analysis was performed using an ultra-performance liquid chromatography-tandem mass spectrometer (UPLC-MS/MS) instrument (Waters Corporation, Milford, MA). For PBAA extraction, samples were hydrolyzed with 6N HCl for 24h at 110°C to remove the free amino acids. After hydrolysis and filtration, 10 µL of the hydrolysate was dried. The pellets were resuspended in Milli-Q purified water containing 13 internal standards and analyzed using the FAA extraction and analysis method. Data processing was performed using the MassLynx data analysis software (TargetLynx XS, Waters, Inc.).

### Popping ability in QPP inbreds

Ten-gram samples of kernels were pooled from five biological replicates of popcorn parents and QPP inbreds. Kernels were popped using an Orville Redenbacher hot air popcorn popper. Total flake volume was measured using a 1000 mL graduated cylinder. Average flake volume of QPP inbreds was compared with that of parental popcorn lines.

### Statistical analysis

Data analysis, visualization, and manipulation were performed using Excel, R (version 4.2.3; 2023-03-15), and packages (ggplot2, cowplot, and dplyr). Data were also checked for normality and homogeneity of variance using the Shapiro-Wilk test and Levene’s test, respectively. Protein concentration, amino acid, and popping volume data were presented as means ± SEM. A one-way analysis of variance (ANOVA), followed by Tukey’s Honestly Significant Difference (HSD) test, was performed to determine significant differences among parental popcorn and the QPP inbreds. Significance levels were presented as *p < 0.05; **p < 0.01; ***p < 0.001.

## Results

### Selection and production of colored QPP inbreds

**Figure 3:**
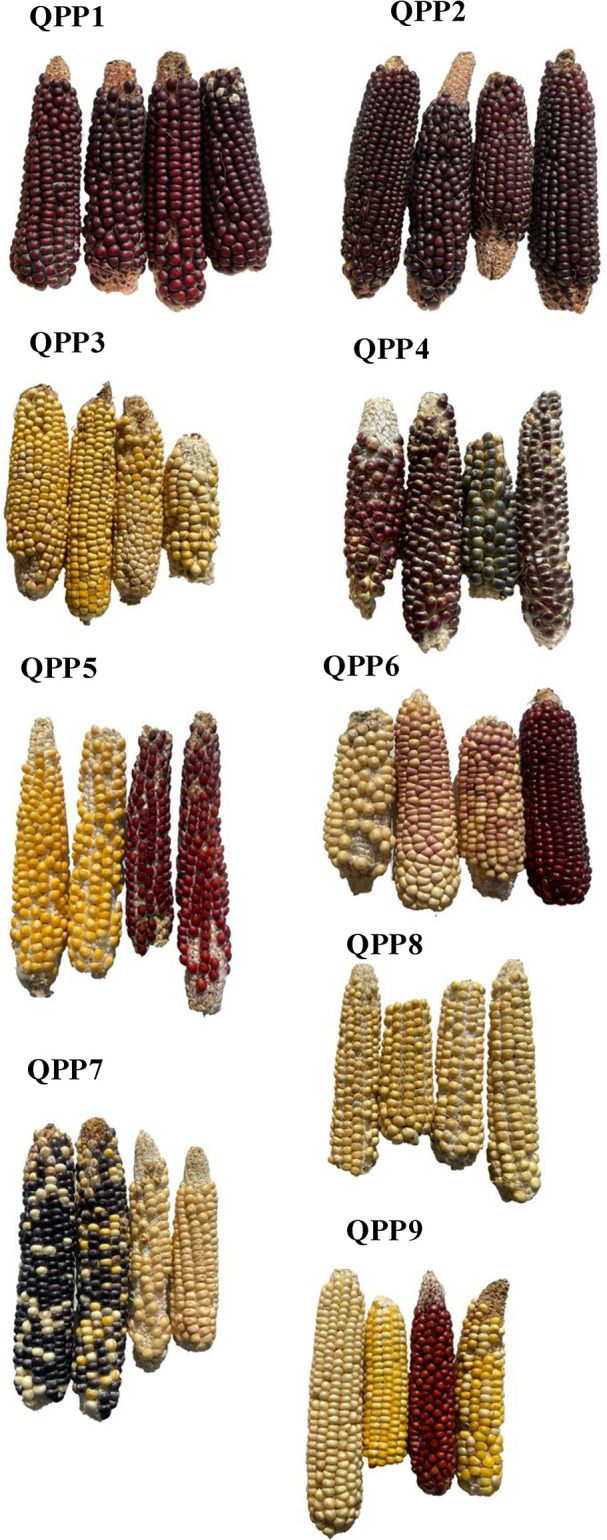
Selection of colored QPP inbreds. Range of kernel colors displayed in BC3F5 colored QPP lines.

In total, nine colored QPP inbreds were produced from the introgression of opaque-2 from the white dent corn QPM into colored popcorns (**Figure 3**). QPP inbreds exhibited different kernel color phenotypes, including red, purple, yellow, and white. The parental popcorns (color donors), such as Cherokee Long Ear Popcorn (CLEP), Cochiti Pueblo Popcorn (CPP), and Glass Gem Popcorn (GG) were highly multi-colored. In contrast, the kernel color phenotype was fixed to a specific color in the colored QPP inbreds as a result of continuous selection for individual kernel color at each generation (**Table 3**). Red pigment accounted for 100%, 98.7%, 40% of color types in QPP1, QPP2, and QPP6 inbreds, respectively. Purple dominated in QPP4 with 86.2%. Yellow was dominant in QPP5 and QPP9 with 92% and 37.5%, respectively, but it was also present across other genotypes except QPP1, QPP4, and QPP6. We also selected for white (lack of pigment) QPP genotypes, and the white genotypes accounted for 43.8%, 68.8%, and 94.2% in QPP3, QPP7, and QPP8, respectively. QPP inbreds 3, 6, 7, 8, and 9 exhibited mixed color types, such as white/yellow and white/purple. These pigments were fixed at the inbred phase compared with earlier generations. Kernel color in the F1 population was highly variable in both nature and uniformity (**Supplementary Figure 1**). The F2 population segregated various kernel color phenotypes, predominantly red, black, and white, with occasional purple phenotypes (**Supplementary Figure 2**). Backcrossing the F2 population to the recurrent popcorn increased kernel color variation in the BC1 population compared with the self-pollinated F3 population (**Supplementary Figure 3**). The BC1F2 population continued to segregate for previous kernel colors, with the addition of more purple kernels (**Supplementary Figure 4**). Backcrossing led to increased color variation; therefore, two additional backcrossing generations were used. Selection pressure favored individual colors; thus, kernel color became increasingly uniform in later generations of QPP selfing.

**Table 3:** Percentage of color types in final QPP inbreds.

Percentage of color type (%) per colored QPP inbred							
QPP inbreds	# Plants	# Red	# Yellow	# White	# White/Purple	# White/yellow	# Purple
QPP1	123	123 (100)	–	–	–	–	–
QPP2	75	74 (98.7)	1 (1.3)	–	–	–	–
QPP3	73	–	35 (48)	32 (43.8)	–	6 (8.2)	–
QPP4	29	–	–	4 (13.8)	–	–	25 (86.2)
QPP5	189	15 (8)	174 (92)	–	–	–	–
QPP6	40	16 (40)	–	9 (22.5)	15 (37.5)	–	–
QPP7	16	–	1 (6.2)	11 (68.8)	3 (18.8)	1 (6.2)	–
QPP8	34	–	2 (5.8)	32 (94.2)	–	–	–
QPP9	120	26 (21.7)	45 (37.5)	14 (11.7)	–	35 (29.1)	–

Total number of plants per QPP genotype. Values in parantheses indicate the percentage of each kernel color within a QPP genotype.

### Confirmation of the *opaque-2* mutation in colored QPP genotypes

**Figure 4:**
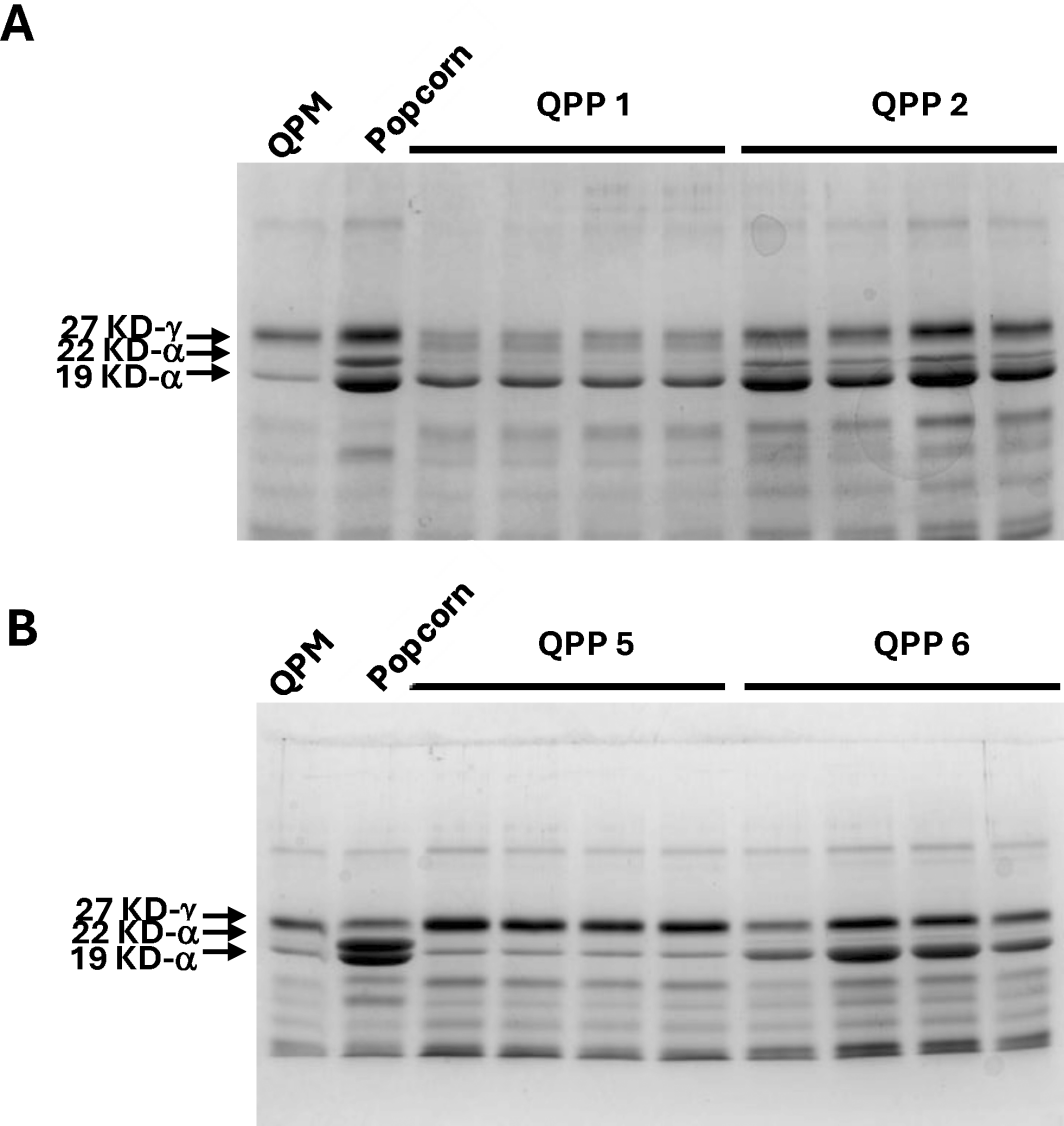
SDS-PAGE confirmation of o2 in the BC3F5 population. **(A)** CML154 introgressions using Red popcorn and Cherokee Long Ear Popcorn (CLEP). **(B)** TX807 introgressions using Red popcorn and CLEP. The o2 confirmations of other QPP inbreds are shown in **Supplementary Figure 5**.

SDS-PAGE analysis of zein fractions confirmed that all nine BC3F5 QPP inbreds exhibited the o2 mutation (**Figure 4**; **Supplementary Figure 5**). The parental popcorns possessed typical α-zein profiles with the pronounced 19-kDa and 22-kDa α zeins. In contrast, a reduction of the 22-kDa α-zein was observed in the QPP2 inbred, while other QPP inbreds displayed a complete knockdown of the 22-kDa α-zein (**Supplementary Table 1**). Additionally, the majority of QPP inbreds, except QPP2, exhibited low 19-kDa α-zeins. The α-zein profiles observed in QPP inbreds were strongly consistent with the α-zein profiles in parental QPM lines, which carry the o2 mutation allele. The BCA total protein analysis revealed that average protein concentrations in colored QPP inbreds ranged between 827 and 1060 μg/mL and were consistent with protein concentrations in parental popcorns, which ranged between 808 and 1076 1060 μg/mL (**Supplementary Figure 6**; **Supplementary Table 2**). The differences in the protein concentrations observed between QPP inbreds and parental popcorn were not significant (p> 0.05) (**Table 4**). Although α-zeins make up a large fraction of total kernel proteins, the reduction of α-zeins did not consequently reduce total protein levels. This conservation of total protein can be attributed to a compensatory mechanism in maize called proteome rebalancing, in which decreased α-zeins trigger increased non-zein protein accumulation. Thus, no change in total protein concentration was reported in the colored QPP inbreds.

**Table 4:** Comparison of BCA total protein between popcorn and QPP inbreds.

Genotypes	Difference	Lower bound	Upper bound	p-value
QPP1-P1	260.239	-9.392	529.871	0.071
QPP2-P2	-60.872	-330.503	208.760	1.000
QPP3-P3	-87.262	-398.606	224.082	1.000
QPP4-P4	73.445	-196.187	343.076	1.000
QPP5-P1	42.182	-227.450	311.813	1.000
QPP6-P2	-112.097	-381.729	157.535	0.987
QPP7-P4	70.598	-199.034	340.229	1.000
QPP8-P5	117.840	-193.504	429.184	0.995
QPP9-QPP6	-115.986	-427.330	195.358	0.996

p-value less 0.05 were considered significant (n=4).

### Visual selection for partially modified QPP genotypes

Opaque2 modifiers (opm) are responsible for kernel vitreousness, which is a critical trait in popcorn. Endosperm modification can be classified into five categories: fully modified (Type 1), partially modified (Types 2, 3, and 4), and unmodified (fully opaque; Type 5). All categories were observed throughout the development of colored QPP inbreds (**Figure 2**; **Supplementary Figures 7**, **8**). Ideally, QPP genotypes exhibiting opm Type 2 were selected because these kernels have good vitreousness while clearly expressing the o2 genotype. Opm Type 1 exhibited higher kernel vitreousness but was more difficult to select because it could be confused with wild-type kernels. The o2 introgressed kernels with white and yellow phenotypes exhibited a unique whitish coloration on the kernel pericarp (**Supplementary Figure 7**). Furthermore, white- and yellow-colored QPP genotypes with opm Type 2 showed residual opaqueness when visualized using a lightbox (**Figure 2**). This enabled identification and selection of o2 introgressed kernels with optimal kernel vitreousness. In contrast, dark colors, such as red, black, and purple, in colored QPP lines obscured the visual o2 phenotype marker and could not be screened using a lightbox. However, endosperm modification could be observed after the kernel pericarp was removed by abrasion (**Supplementary Figure 8**). This approach allowed the visual identification of the relative amounts of vitreous and opaque endosperm, revealing the o2 modifier status and ensuring that colored QPP genotypes with desired opm could be confidently identified. colored lines among from QPP1 through QPP9 were evaluated using this method, whereas yellow and white lines were assessed using the lightbox.

### Amino acid profiles in whole QPP kernels and popped QPP flakes

**Figure 5:**
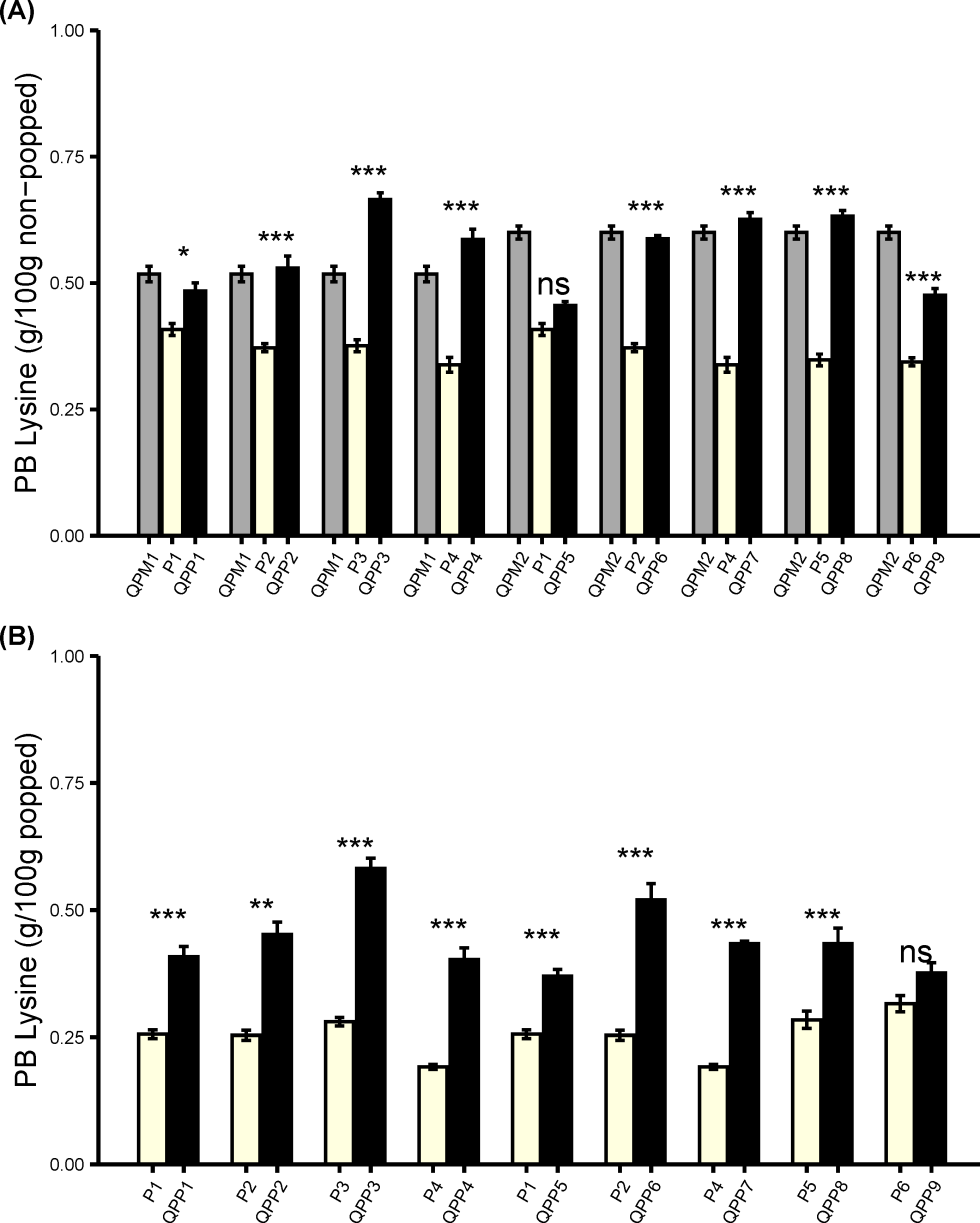
Protein-bound lysine content in QPP inbreds. **(A)** Non-popped QPP **(B)** Popped QPP. Data are presented as means ± SEM for n=5 biological replicates. One-way ANOVA was performed, followed by Tukey’s HSD test to determine differences between parental popcorn (control; light yellow) and QPP inbreds (treatment; black). Significance levels are presented as *p < 0.05; **p < 0.01; ***p < 0.001.

**Figure 6:**
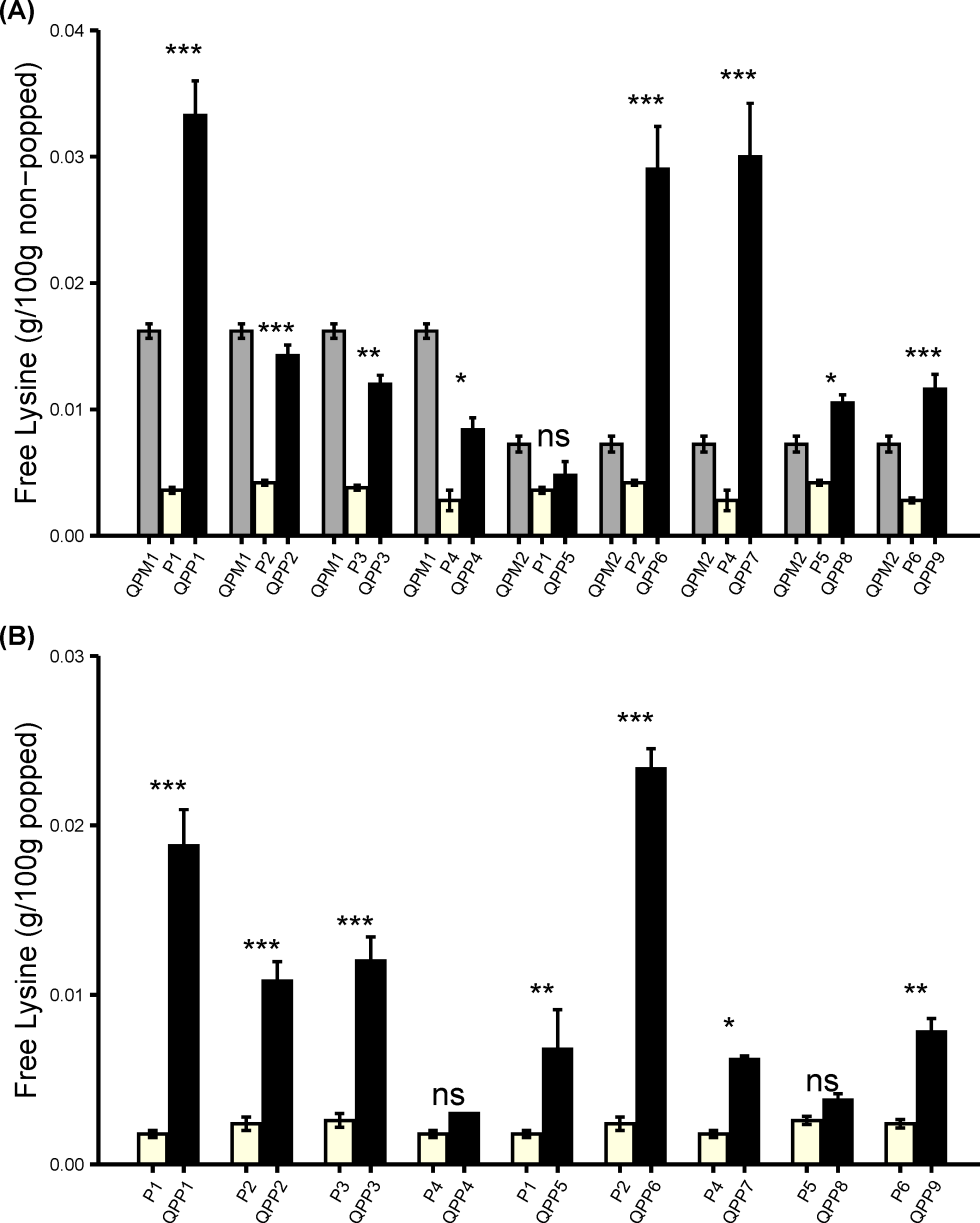
Free lysine content in QPP inbreds. **(A)** Non-popped QPP **(B)** Popped QPP. Data are presented as means ± SEM for n = 4 biological replicates. One-way ANOVA was performed, followed by Tukey’s HSD test to determine differences between parental popcorn (control; light yellow) and QPP inbreds (treatment; black). Significance levels are presented as *p < 0.05; **p < 0.01; ***p < 0.001.

To determine whether o2 introgression into the popcorn germplasm increased lysine content in colored QPP inbreds, PBAA and FAA contents were profiled in the normal popcorn parental lines and the nine colored QPP inbreds (**Figures 5**, **6**). The resulting QPP inbreds had lower lysine-devoid α-zeins than the normal popcorns. The total protein concentrations of both normal popcorn and QPP inbreds were nearly the same, suggesting that the lysine-rich non-zein proteins were increased via the proteome rebalancing effect. I addition to lysine, 15 other amino acids (Ala, Arg, Asx, Glx, Gly, His, Ile, Leu, Met, Phe, Pro, Ser, Thr, Tyr, and Val) were measured to further assess the broader impact of o2 on the overall amino acid profile of the QPP relative to the normal popcorn (**Supplementary Figures 10**–**24**).

The results showed that the protein-bound (PB) lysine increased in both whole kernels and popped flakes across all nine colored QPP inbreds (**Figure 5**). The PB lysine increase was significant in nearly all colored QPP inbreds except QPP5 in whole kernels (**Figure 5A**) and QPP9 in popped flakes (**Figure 5B**). Although PB lysine increased in both whole kernel and popped flake QPPs, PB lysine content was lower in popped flake QPPs than in their corresponding whole kernel QPPs (**Supplementary Tables 3**, **4**). However, the PB lysine increase ratio between QPPs and the corresponding popcorn parent was smaller in whole kernel form than in popped flake form (**Supplementary Table 5**). Specifically, the PB lysine ratio between QPP and popcorn parent ranged from 1.12-fold (QPP5 and P1) to 1.87-fold (QPP7 and P4) in whole kernel form, compared with 1.2-fold (QPP9 and P6) to 2.27-fold (QPP7 and QPP4) in popped flakes. This result suggests that, after popping, QPP inbreds retained higher PB lysine levels than parental popcorns, indicating that PB lysine in colored QPP is more resilient to heat than in normal colored popcorn parents. Similarly to PB lysine, the free lysine increased across all colored QPPs compared with the parental popcorns in both whole kernel and popped flake forms (**Figure 6**). The free lysine increase was significant in nearly all colored QPPs except QPP5 in whole kernels (**Figure 6A**) as opposed to QPP4 and QPP8 in popped flakes (**Figure 6B**). Free lysine levels in colored QPPs were higher in whole kernel form relative to the corresponding popped flake form, except in QPP3 and QPP5, where free lysine in whole kernels relative to popped flakes were equal and lower, respectively (**Supplementary Table 6**). The ratio ranged from 1.36-fold (whole kernel QPP6/popped flake QPP6) to 4.85-fold (whole kernel QPP7/popped flake QPP7) (**Supplementary Table 7**). However, the ratio between colored QPPs and parental popcorns was higher in popped flakes than in whole kernels for certain inbreds, such as QPP1, QPP3, and QPP5 (**Supplementary Table 8**). In contrast to PB lysine, six of nine colored QPP inbreds lost more free lysine when popped compared with parental popcorn lines. Overall, o2 introgression into colored popcorn germplasm clearly increased its protein-bound free lysine content in both whole kernel and popped flake forms. Similarly to lysine, whole kernel and popped flake amino acids content were also measured in 15 other amino acids (**Supplementary Figures 9**–**24**). The results showed that protein-bound amino acids (Hist, Arg, Asp, and Gly) were significantly increased, whereas others while (Ile, Leu, Met, Thr, Val, Pro, Glx, Tyr, Ser, and Ala) were significantly decreased in the majority of colored QPPs, regardless of whether the kernel was whole or popped. In contrast, free amino acids were significantly increased in nearly all colored QPPs, with only a few QPPs showing no significant change for any of these amino acids.

### Evaluation of popping ability in colored QPP inbreds

**Figure 7:**
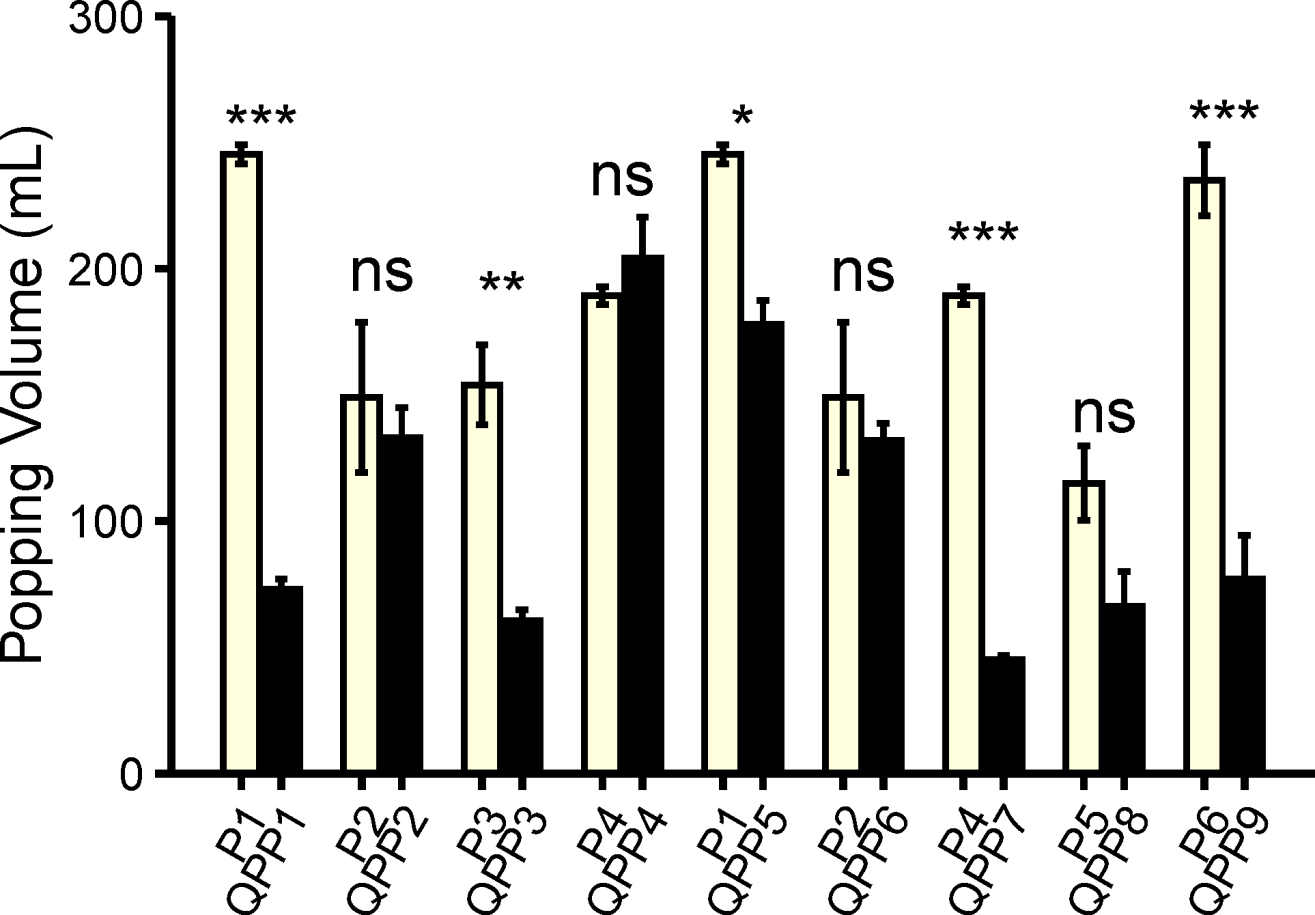
Popping volume of QPP inbreds. Data are presented as means ± SEM for n = 5 biological replicates. One-way ANOVA was performed, followed by Tukey’s HSD test to determine differences between parental popcorn (control; light yellow) and QPP inbreds (treatment; black). Significance levels were presented as *p < 0.05; **p < 0.01; ***p < 0.001.

To assess the effects of the o2 mutation and o2 modifiers on the popping quality of the colored QPP inbreds, kernel flake volume was measured (**Figure 7**; **Supplementary Figures 25**, **26**). Five colored QPP inbreds, namely, QPP1, QPP3, QPP5, QPP7, and QPP9, had significantly smaller average flake volume than the normal popcorn parents (p < 0.05) (**Tables 5**, **6**). The average kernel flake volume reduction in these QPP inbreds ranged between 2- and 4-fold relative to the normal popcorn (**Table 6**). In contrast, QPP2, QPP4, QPP6, and QPP8 showed relatively equal flake volume compared with their respective popcorn parents. Ranking the QPP inbreds by their overall popping ability relative to their respective parental popcorn resulted in the following order: QPP4, QPP2, QPP6, QPP5, QPP8, QPP3, QPP9, QPP1, and QPP7. The average flake volume of QPP inbreds was consistently low, with QPP7 showing the lowest flake volume of 45 mL in compared with its P4 popcorn parent at 189 mL. In contrast, QPP4, which was derived from the same popcorn parent as QPP7, had a flake volume of 204 mL. The difference between the QPP4 and QPP7 inbreds is that they were crossed to different QPM parents, namely CML154 and Tx807, respectively. Similarly, QPP1 and QPP5 were derived from the same P1 popcorn parent but crossed to CML154 and Tx807, respectively. However, QPP1 had a lower flake volume of 74 mL compared with QPP5 at 178 mL. Taken together, these results indicate that the QPM parent was not a determining factor in flake volume variation among QPP inbreds. In general, kernel flake volumes varied markedly among QPP introgressions and among biological replicates of the same QPP introgression.

**Table 5:** Preliminary popping evaluation of QPP inbreds and parental popcorns.

	Popping volume (mL)						
Genotype	Ear # 1	Ear# 2	Ear # 3	Ear # 4	Ear # 5	Mean	sd
P1	235	257	240	250	245	245	9
QPP1	85	70	65	85*	65	74	35
QPP5	170	155	165	195	205*	178	3
P2	185	240	70	105	145	149	10
QPP2	115	120	130	120	180*	133	10
QPP6	110	140	150*	130	130	132	33
P3	145	195	135	110	185	154	21
QPP3	70	60	70*	55	48	60.6	8
P4	195	198	190	186	178	189	67
QPP4	159	257*	217	188	200	204	36
QPP7	48*	44	40	46	48	45	30
P5	145	95	100	80	155	115	27
QPP8	120*	62	48	50	52	66	32
P6	265	270	210	200	230	235	15
QPP9	43	48	95	135*	65	77	38

QPP inbreds marked with * were selected for the QPP hybrid production.

**Table 6:** Flake volume ratio and average comparison between parental popcorn and respective QPP inbreds.

Genotype	P/QPP ratio	Difference	Lower bound	Upper bound	p-value
QPP1-P1	3.3	-172.4	-238.7	-106.1	0.000
QPP2-P2	1.1	-16.0	-82.3	50.3	1.000
QPP3-P3	2.5	-93.4	-159.7	-27.1	0.001
QPP4-P4	0.9	14.8	-51.5	81.1	1.000
QPP5-P1	1.4	-67.4	-133.7	-1.1	0.043
QPP6-P2	1.1	-17.0	-83.3	49.3	1.000
QPP7-P4	4.2	-144.2	-210.5	-77.9	0.000
QPP8-P5	1.7	-48.6	-114.9	17.7	0.396
QPP9-P6	3.0	-157.8	-224.1	-91.5	0.000

P/QPP ratio of flake volume (n=5). p-value less 0.05 were considered significant (n=5).

## Discussion

In this study, our objective was to breed quality protein traits into colored popcorn germplasm. A total of nine colored quality protein popcorn inbreds were produced. The results showed that protein-bound and free lysine contents significantly increased across nearly all colored QPP inbreds in both whole kernel and popped flake forms. Such increase in lysine content are attributable to the consistently low alpha zeins, especially the 22-kDa α-zein fractions, reported in the colored QPPs. Maize endosperm storage proteins consist largely of the zein protein, of which α-zein accounts for approximately 70% (Yue et al., 2014). These α-zeins lack lysine, resulting in limited lysine content in normal maize, including popcorn. Maize endosperm proteins are known to compensate for the reduction of α-zeins by accumulating more non-zeins through a process called proteome rebalancing (Wu and Messing, 2014). Here, in a similar fashion, the reduction of the 22-kDa α-zein observed in the colored QPP inbreds resulted in an increase of the non-zein proteins. It is evident that kernel total protein concentrations across all nine colored QPPs and normal popcorn parental lines remained relatively equivalent, ranging between 827-1069 μg/mL and 808-1076 μg/mL, respectively. This suggests that lysine-rich non-zeins increasingly accumulated in the QPPs, leading to the reported significantly higher lysine content in colored QPP inbreds (p<0.05). The increase in lysine levels implies that the level of tryptophan also increased. Due to its unstable structure, tryptophan degrades during the acid hydrolysis required to break down the protein-bound amino acids and requires a separate extraction method. However, tryptophan has a high correlation with lysine, maintaining an approximate 4:1 lysine-to-tryptophan ratio (Hernandez and Bates, 1969). Thus, estimating of tryptophan concentration based on lysine concentration is routine.

Although breeding for improved protein quality in colored popcorn improved the lysine and tryptophan contents in the resultant colored QPPs, collateral changes in other amino acids were observed, as indicated by consistently low QPP/popcorn rations (< 1) (**Supplementary Table 5**). Consistent with our findings, the negative correlation between the o2 and some amino acids (Leu, Met, Thr, Val, Pro, Glx, Tyr, Ser, and Ala) in the QPPs have been previously reported (Hartings et al., 2011; Ren et al., 2018). However, these adverse effects of o2 were primarily evident in the protein-bound fractions. In contrast, the free fractions of these amino acids were significantly increased across the majority of the colored QPPs (**Supplementary Figures 9**–**24**). The increase in the free amino acids might be directly linked to the reduced expression of the OPAQUE-2 (O2) gene due to the mutation. The O2 transcription factor positively regulates enzymes, such as lysine ketoglutarate reductase (LKR) and aspartate kinase 2 (Ask2), which catabolize free amino acids in maize (Wang et al., 2001). Thus, reduced O2 function results in reduced activity of these catabolic enzymes, leading to increased accumulation of free amino acids, with the ratio of concentrations QPP/P > 1 (**Supplementary Table 8**). However, protein-bound amino acids have much higher concentrations and therefore contribute more significantly to overall kernel amino acid levels. The results indicated that the free amino acid fractions were at least 10-fold lower than the protein-bound fractions. The increases in free amino acids are likely not sufficient to compensate for the reduction of the corresponding protein-bound amino acids.

We observed a slight decline of amino acids profiles after popping in both colored QPPs and normal colored popcorn parental lines (**Supplementary Tables 4**, **6**). However, the protein-bound and free amino acids remained relatively high in the QPPs compared with the parental popcorns. Similar observations were previously shown in which QPP hybrids were popped using different methods: air, oil, and microwave (Parsons et al., 2020). That study found that amino acids levels were reduced in the QPP hybrids and popcorn parents, but the QPP hybrids retained higher levels of amino acids compared with the popcorn parents, particularly with the air-popping method. In general, the stability of amino acids declines when subjected to heat. Additionally, the reduction of amino acids has been reported in other popped grains, such as kiwicha and quinoa (Paucar-Menacho et al., 2018). We cannot explain the reason why colored QPPs maintained higher amino acids than popcorn parents after popping. However, it is possible that the o2 mutation or even kernel color might have contributed to some extent to the conservation of either protein-bound or free amino acids.

Apart from the reported improvement of lysine content and overall protein quality in the maize o2 mutants, the QPM introgression introduces collateral defects in kernel endosperm vitreousness by increasing the soft (opaque) endosperm fraction. In popcorn, kernel vitreousness is one of the major factors determining popping quality. The popping quality declined significantly, by 2- to 4-fold, in five colored QPP inbreds (p<0.05) (**Figure 7**; **Table 6**), despite having been backcrossed three times to the recurrent parental popcorn. In previous QPP studies, in addition to backcrossing the QPP to the recurrent popcorn parent, the o2 endosperm modifiers responsible for the kernel vitreousness were selected (Ren et al., 2018). Furthermore, significant improvements in popping quality were observed in BC3 QPP hybrids compared with their respective BC2 lines (Parsons et al., 2021a). However, those studies were performed only with elite yellow and white colored popcorns, where endosperm modification is most likely easier to detect using a lightbox or by visual identification of the o2 kernel phenotype. The o2 mutation cast a whitish opaque spot on the kernel pericarp, indicating the presence of o2 mutation and allowing visualization of endosperm modification levels (**Supplementary Figures 7**, **8**). With dark colored kernels, such as red, blue, and purple, reported in the colored QPPs, lightbox screening of vitreousness and opacity were not possible because kernel pigments obscured the o2 phenotype; therefore, manual exposure of the endosperm was required to observe the opaque endosperm modification (**Supplementary Figure 9**). The selection of desired endosperm modification was more difficult and uncertain in colored QPPs. Without the ability to use lightbox selection, our strategy was to include all inbreds that could potentially contribute to acceptable popping in the hybrids produced in the next phase of the project.

In this study, confirmation and selection of o2 genotypes and opaque endosperm modification relied mainly on the SDS-PAGE analysis and physical identification of the kernel o2 phenotypes. Common o2 polymorphic markers (umc1066, phi057, phi112) (Babu et al., 2015) were tried to genotype the o2 allele and the 27-kDa γ-zein duplication (o7o7) in the early germinating QPP lines. However, the genetic markers did not yield conclusive results with the o2 donor used in this study since they did not reliably show the expected polymorphism. The selection for o2 and the 27-kDa γ-zein duplication (Liu et al., 2016) relied solely on visual selection followed by SDS-PAGE rather than DNA markers. Several studies have linked the expression and protein accumulation of 27-kDa γ-zein to the endosperm modification in QPM lines (Geetha et al., 1991). Although 27-kDa γ-zein is a highly reliable protein marker for QPM, unknown endosperm modifiers necessitate continued visual kernel screening. Maintaining kernel vitreousness should remain a priority for future popcorn breeding programs. The generated colored QPP inbreds are currently being used for hybrid development. Colored QPP hybrids will ensure optimal endosperm modification to overcome reduced kernel flake and promote hard kernel agronomics essential for long-term storage and handling. The evaluation of the QPP hybrids will involve multi-location yield trials to select and confirm superior lines with increased plant and seed health to prevent vulnerability on the market and consumer preferences.

In summary, this work produced nine colored quality protein popcorn inbreds. We demonstrated that breeding the o2 in colored popcorn germplasm reduced lysine-devoid alpha zeins, leading to increased accumulation of lysine-rich proteins, significantly improving lysine content in both whole kernels and popped flakes. The QPP introgressions also showed substantial variation in kernel flake volume, which indicates varying levels of endosperm modification, emphasizing the need to optimize methods to identify opm with certainty to overcome the challenges of maintaining optimal popcorn qualities, such as hard endosperm and maximum kernel expansion. The average popping quality declined in some QPP inbreds, although some individual lines showed no significant difference to the respective popcorn parents. Kernel pigmentation in colored QPP further complicated the complex process of identifying modifier genes by obscuring endosperm modification. Despite this challenge, we demonstrated that the phenotypic selection of the opaque endosperm modifiers, even with the obscuring kernel color, recovered the popping ability in certain lines, restoring to levels of the parental popcorn lines. Although some inbreds showed reduced pop volume, hybrid production and the resultant heterosis will likely yield improved flake volume and flake morphology phenotypes, as was observed in a previous study (Parsons et al., 2021a). Furthermore, elite proprietary QPP hybrids showed promising improvements in consumer taste scores (Parsons et al., 2021b) and prebiotic microbiome effects (Korth et al, 2022). The more diverse popcorn germplasm used in the current study will likely produce even more diversity in taste and nutritional properties. For these reasons, and to avoid constraints of proprietary germplasm, the development of new inbreds and hybrids is warranted. This work contributes to broader efforts towards nutritionally enhanced staple and snack foods, positioning Quality Protein Popcorn as a promising candidate in both categories.

## Data Availability

The original contributions presented in the study are included in the article/**Supplementary Material**. Further inquiries can be directed to the corresponding author.

## Supplementary material

Supplementary Figure 1 Kernel color introgression in F1 QPP lines.

Supplementary Figure 2 Kernel color introgression in F2 QPP lines.

Supplementary Figure 3 Kernel color introgression in F3 and BC1 QPP lines.

Supplementary Figure 4 Kernel color introgression in BC1F2 QPP lines

Supplementary Figure 5 SDS-PAGE confirmation of o2 in BC3F5 colored QPP. **(A)** CML154 introgression using Cochiti Pueblo Popcorn and Black Jewel Popcorn. **(B)** TX807 introgressions using Black Jewel Popcorn and Glass Gem Popcorn. **(C)** TX807 introgression using Mini Blue Popcorn.

Supplementary Figure 6 BCA total protein concentration in QPM, parental popcorn, and QPP inbreds. Results were shown as Means ± SEM for n =4 biological replicates.

Supplementary Figure 7 Identification of opaque endosperm modifiers in yellow and white kernelled QPP inbreds.

Supplementary Figure 8 Identification of opaque endosperm modifiers in dark colored QPP lines.

Supplementary Figure 9 Free tryptophan content in QPP inbreds. **(A)** non-popped QPP **(B)** popped QPP. Data were presented as Means ± SEM for n=4 biological replicates. Data were presented as Means ± SEM for n=5 biological replicates. One-way ANOVA was performed followed by Tukey HSD to determine differences between parental popcorn (control; light yellow) and QPP inbreds (treatment; black). Significance levels were presented as *p < 0.05; **p < 0.01; ***p < 0.001.

Supplementary Figure 10 Histidine content in QPP inbreds. **(A)** PBAA in non-popped lines **(B)** PBAA in popped lines. **(C)** FAA in non-popped lines **(D)** FAA in popped lines.

Supplementary Figure 11 Isoleucine content in QPP inbreds. **(A)** PBAA in non-popped lines **(B)** PBAA in popped lines. **(C)** FAA in non-popped lines **(D)** FAA in popped lines.

Supplementary Figure 12 Leucine content in QPP inbreds. **(A)** PBAA in non-popped lines **(B)** PBAA in popped lines. **(C)** FAA in non-popped lines **(D)** FAA in popped lines.

Supplementary Figure 13 Methionine content in QPP inbreds. **(A)** PBAA in non-popped lines **(B)** PBAA in popped lines. **(C)** FAA in non-popped lines **(D)** FAA in popped lines.

Supplementary Figure 14 Phenylalanine content in QPP inbreds. **(A)** PBAA in non-popped lines **(B)** PBAA in popped lines. **(C)** FAA in non-popped lines **(D)** FAA in popped lines.

Supplementary Figure 15 Threonine content in QPP inbreds. **(A)** PBAA in non-popped lines **(B)** PBAA in popped lines. **(C)** FAA in non-popped lines **(D)** FAA in popped lines.

Supplementary Figure 16 Valine content in QPP inbreds. **(A)** PBAA in non-popped lines **(B)** PBAA in popped lines. **(C)** FAA in non-popped lines **(D)** FAA in popped lines.

Supplementary Figure 17 Proline content in QPP inbreds. **(A)** PBAA in non-popped lines **(B)** PBAA in popped lines. **(C)** FAA in non-popped lines **(D)** FAA in popped lines.

Supplementary Figure 18 Glutamine content in QPP inbreds. **(A)** PBAA in non-popped lines **(B)** PBAA in popped lines. **(C)** FAA in non-popped lines **(D)** FAA in popped lines.

Supplementary Figure 19 Tyrosine content in QPP inbreds. **(A)** PBAA in non-popped lines **(B)** PBAA in popped lines. **(C)** FAA in non-popped lines **(D)** FAA in popped lines.

Supplementary Figure 20 Serine content in QPP inbreds. **(A)** PBAA in non-popped lines **(B)** PBAA in popped lines. **(C)** FAA in non-popped lines **(D)** FAA in popped lines.

Supplementary Figure 21 Alanine content in QPP inbreds. **(A)** PBAA in non-popped lines **(B)** PBAA in popped lines. **(C)** FAA in non-popped lines **(D)** FAA in popped lines.

Supplementary Figure 22 Arginine content in QPP inbreds. **(A)** PBAA in non-popped lines **(B)** PBAA in popped lines. **(C)** FAA in non-popped lines **(D)** FAA in popped lines.

Supplementary Figure 23 Asparagine content in QPP inbreds. **(A)** PBAA in non-popped lines **(B)** PBAA in popped lines. **(C)** FAA in non-popped lines **(D)** FAA in popped lines.

Supplementary Figure 24 Glycine content in QPP inbreds. **(A)** PBAA in non-popped lines **(B)** PBAA in popped lines. **(C)** FAA in non-popped lines **(D)** FAA in popped lines.

Supplementary Figure 25 Relative flake size of parental colored popcorn and colored QPP.

Supplementary Figure 26 Relative flake size of parental colored popcorn and colored QPP.

Supplementary Table 1 Description of zein levels in colored QPP inbreds.

Supplementary Table 2 Total protein concentrations in QPM, popcorn, and colored QPP inbreds lines

Supplementary Table 3 Mean PBAA values of parental popcorns and colored QPP inbreds.

Supplementary Table 4 Non-popped/popped ratio values of PBAA fractions in parental popcorn and colored QPP inbreds.

Supplementary Table 5 QPP/P ratios of PBAA in popped and non-popped lines.

Supplementary Table 6 Mean FAA values of parental popcorns and colored QPP inbreds.

Supplementary Table 7 Non-popped/popped ratio values of FAA fractions in parental popcorn and colored QPP inbreds.

Supplementary Table 8 QPP/P ratios of FAA in popped and non-popped lines.
