# Supplementary material for: Breeding for colored quality protein popcorn with improved amino acid composition
Source: Front Plant Sci. 2026 May 25;17:1844370. doi: 10.3389/fpls.2026.1844370 (PMC13243419; doi:10.3389/fpls.2026.1844370)

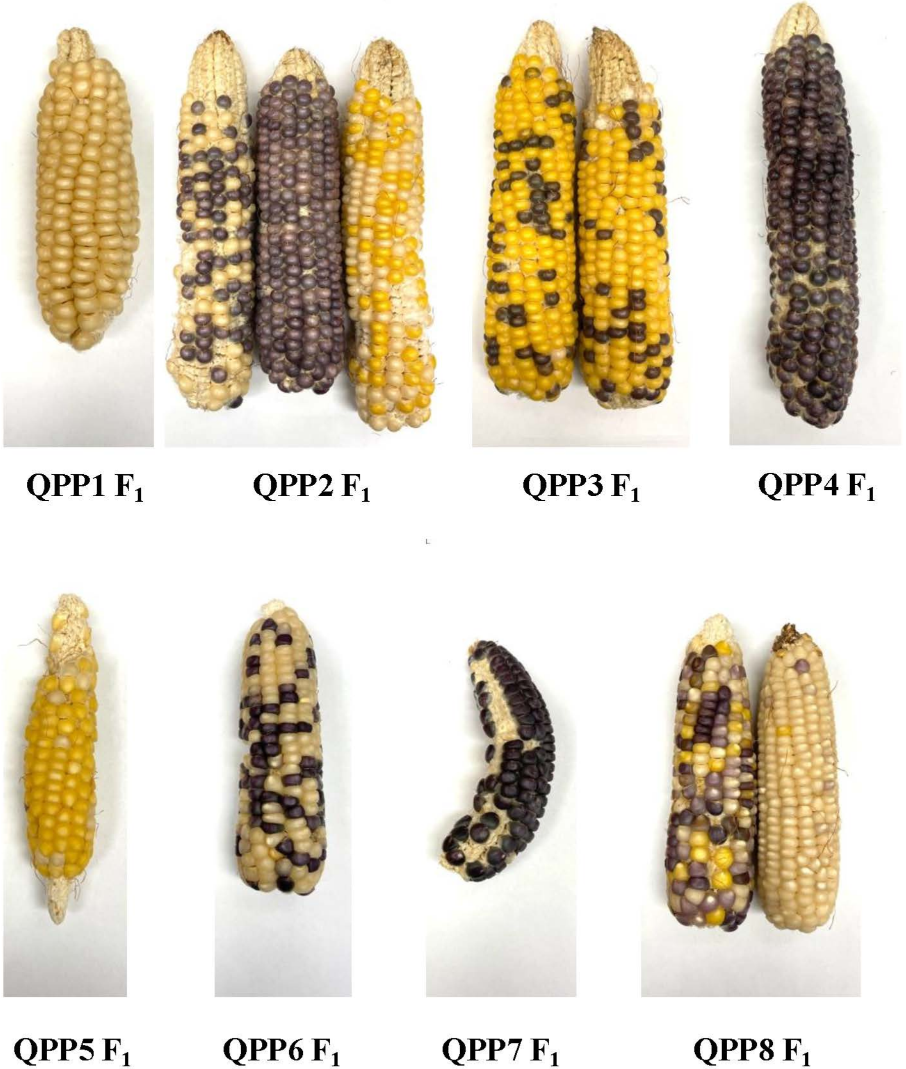

Supplementary Figure S1| Kernel color introgression in F1 QPP lines

Supplementary Figure S2| Kernel color introgression in F2 QPP lines

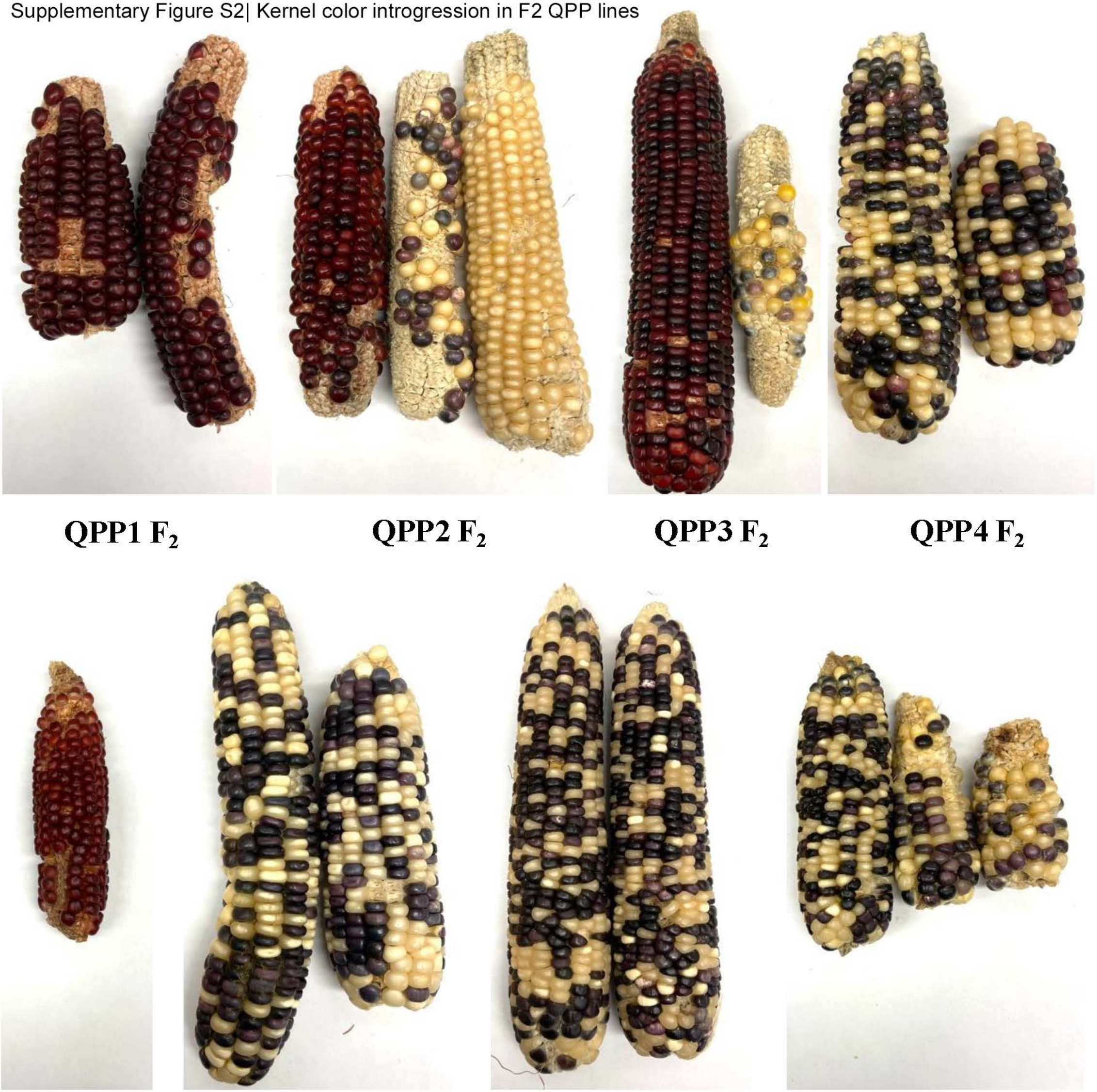

QPP1 F_2 QPP2 F_2 QPP3 F_2 QPP4 F_2

QPP5 F_2 QPP6 F_2 QPP7 F_2 QPP8 F_2

Supplementary Figure S3| Kernel color introgression in F3 and BC1 QPP lines

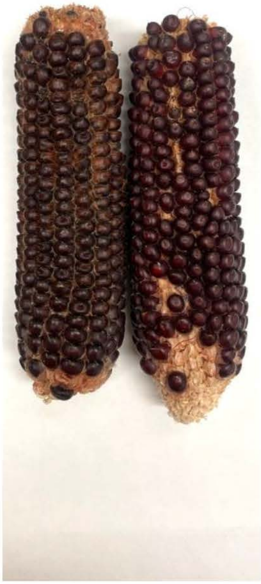

QPP1 F_3 BC_1

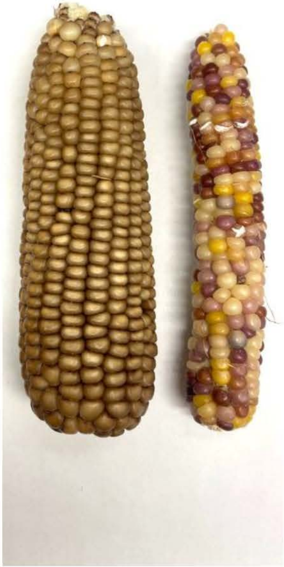

QPP2 F_3 BC_1

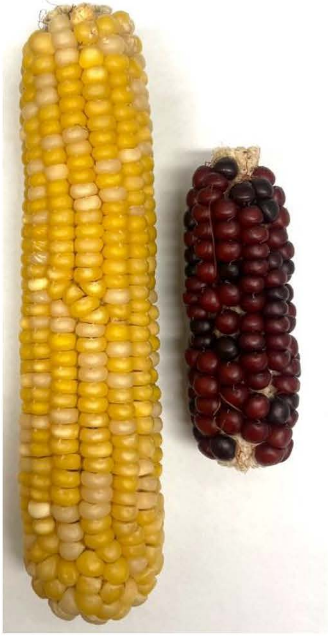

QPP3 F_3 BC_1

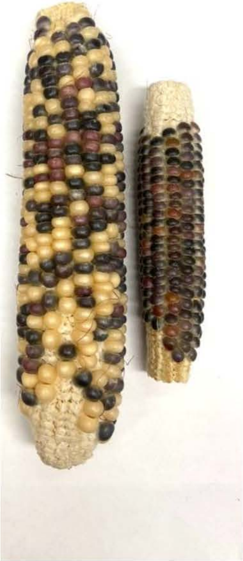

QPP4 F_3 BC_1

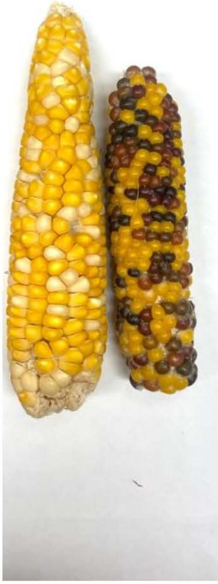

QPP5 F_3 BC_1

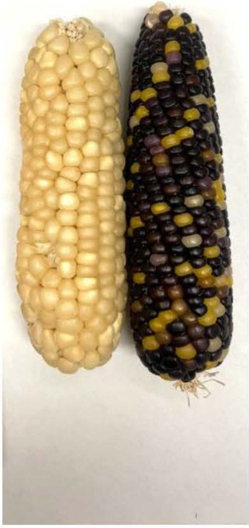

QPP6 F_3 BC_1

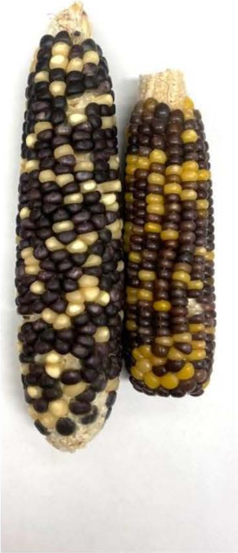

QPP7 F_3 BC_1

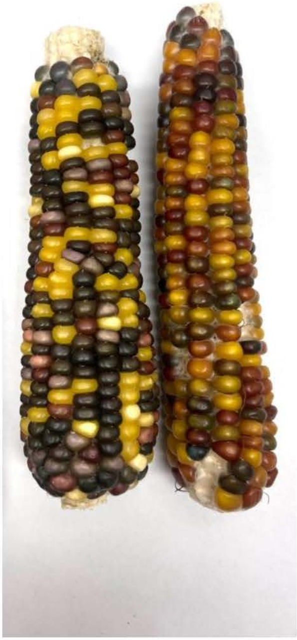

QPP8 F_3 BC_1

Supplementary Figure S4| Kernel color introgression in BC1F2 QPP lines

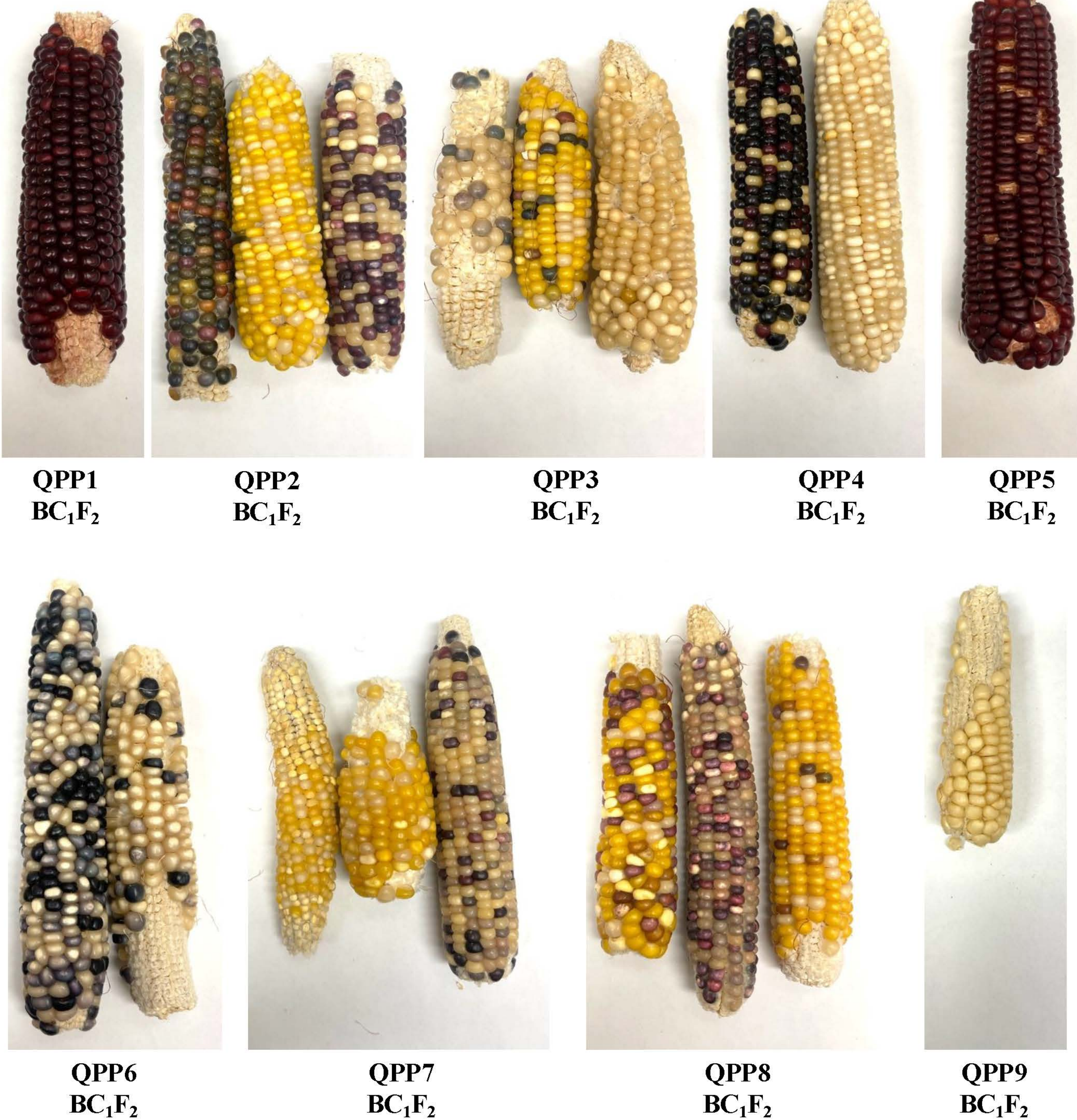

Supplementary Figure S5| SDS-PAGE confirmation of o2 in BC3F5 colored QPP. (A) CML154 introgression using Cochiti Pueblo Popcorn and Black Jewel Popcorn. (B) TX807 introgressions using Black Jewel Popcorn and Glass Gem Popcorn. (C) TX807 introgression using Mini Blue Popcorn.

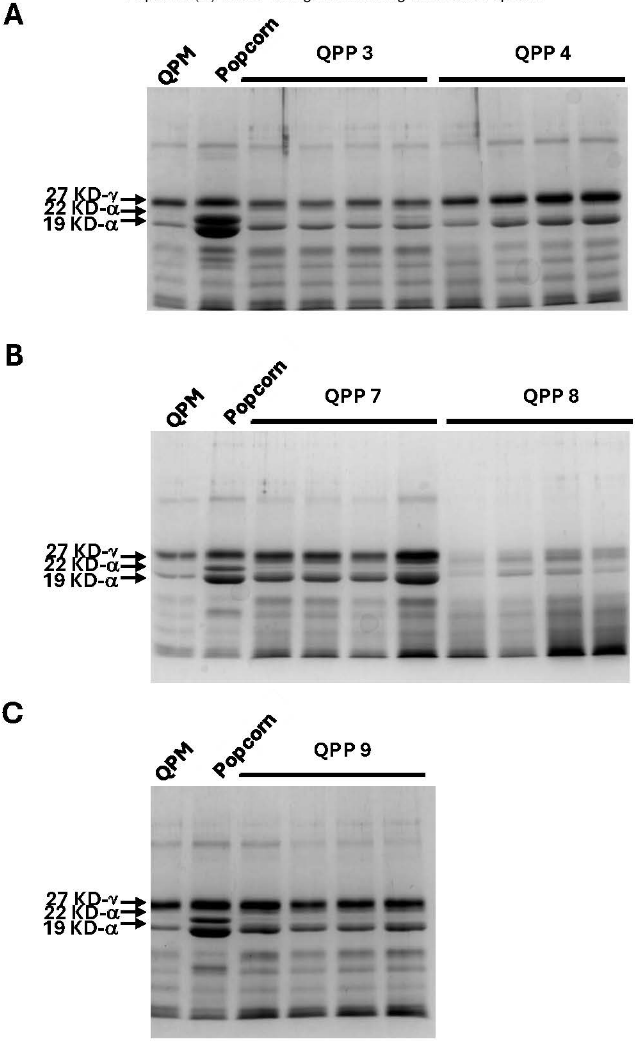

Supplementary Figure S6| BCA total protein concentration in QPM, parental popcorn, and QPP inbreds. Results were shown as Means ± SEM for n = 4 biological replicates.

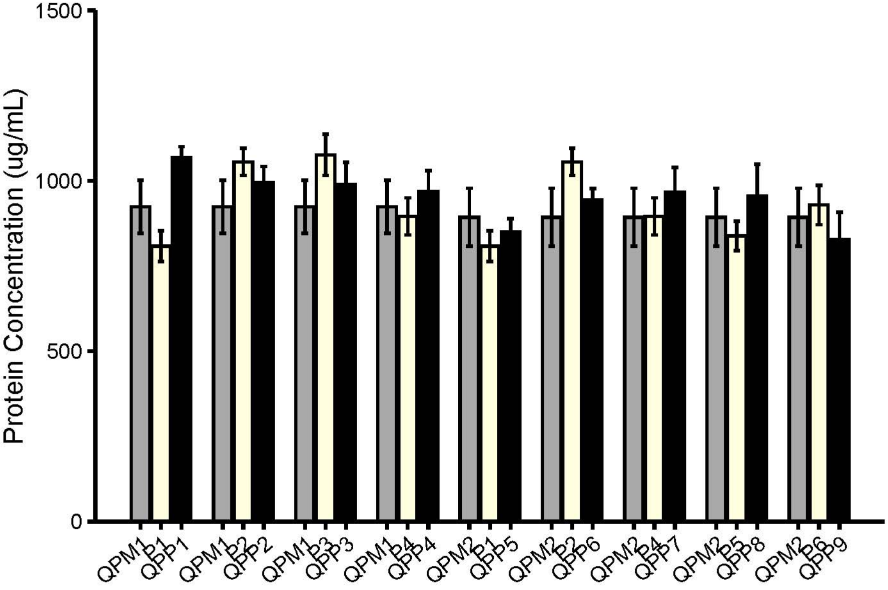

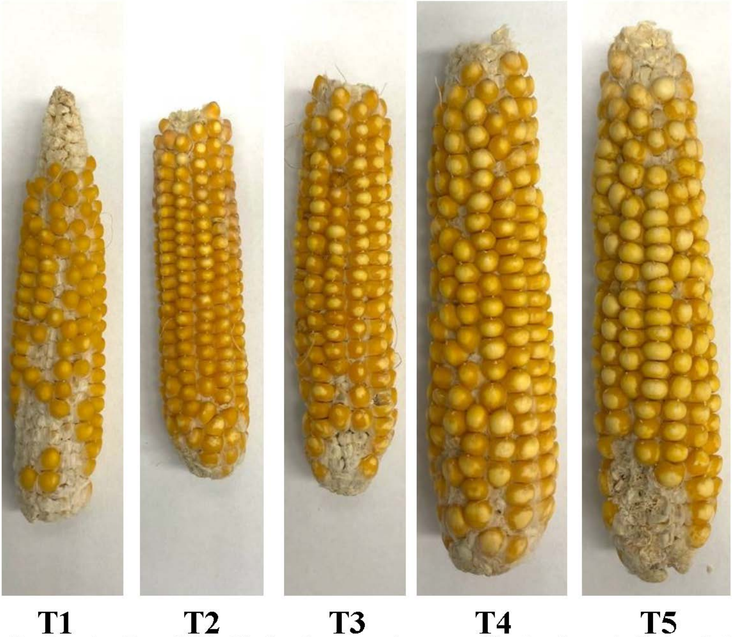

Supplementary Figure S7| Identification of opaque endosperm modifiers in yellow and white kernelled QPP inbreds

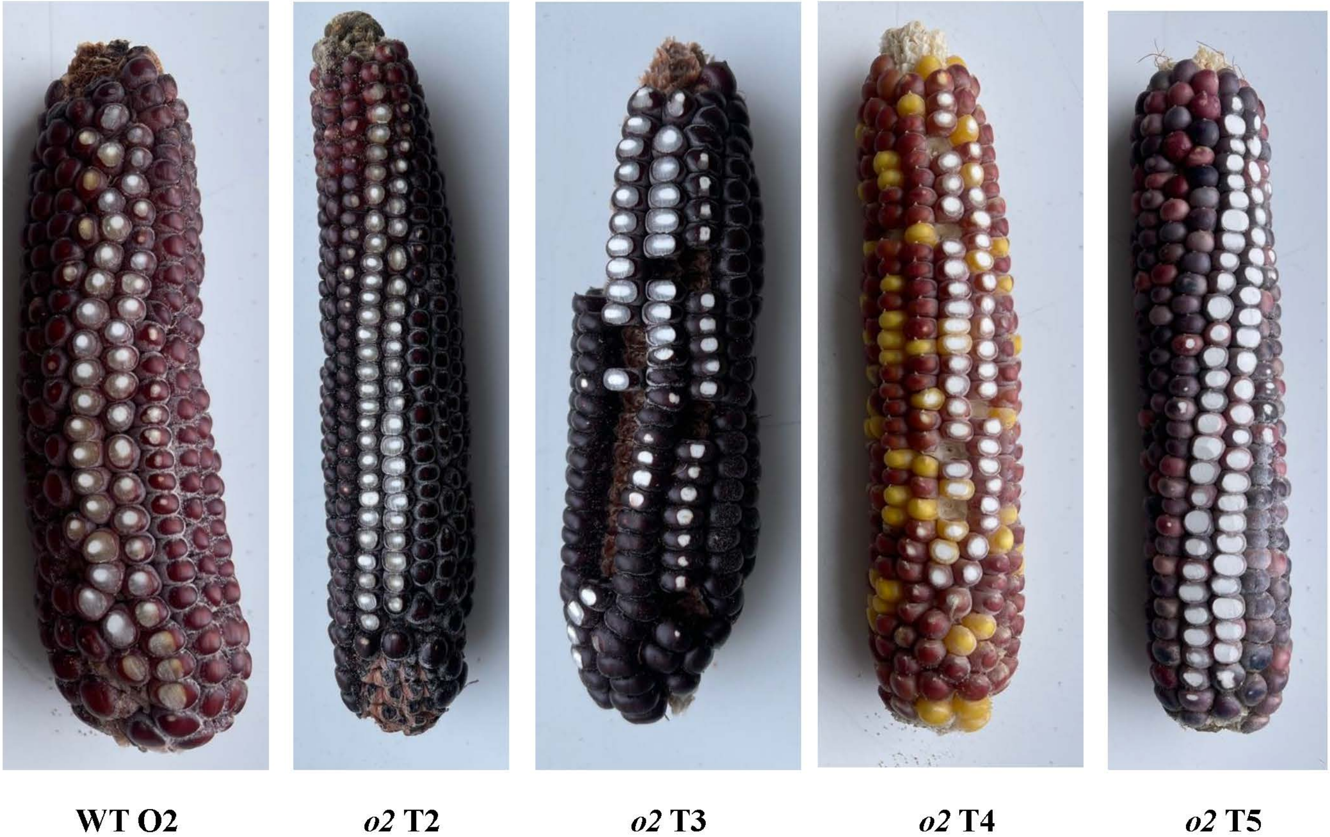

Supplementary Figure S8| Identification of opaque endosperm modifiers in dark colored QPP lines.

Supplementary Figure S9| Free tryptophan content in QPP inbreds.
(A) non-popped QPP (B) popped QPP. Data were presented as Means ± SEM for n = 4 biological replicates. Data were presented as Means ± SEM for n = 5 biological replicates. One-way ANOVA was performed followed by Tukey HSD to determine differences between parental popcorn (control; light yellow) and QPP inbreds (treatment; black). Significance levels were presented as *p < 0.05; **p < 0.01; ***p < 0.001

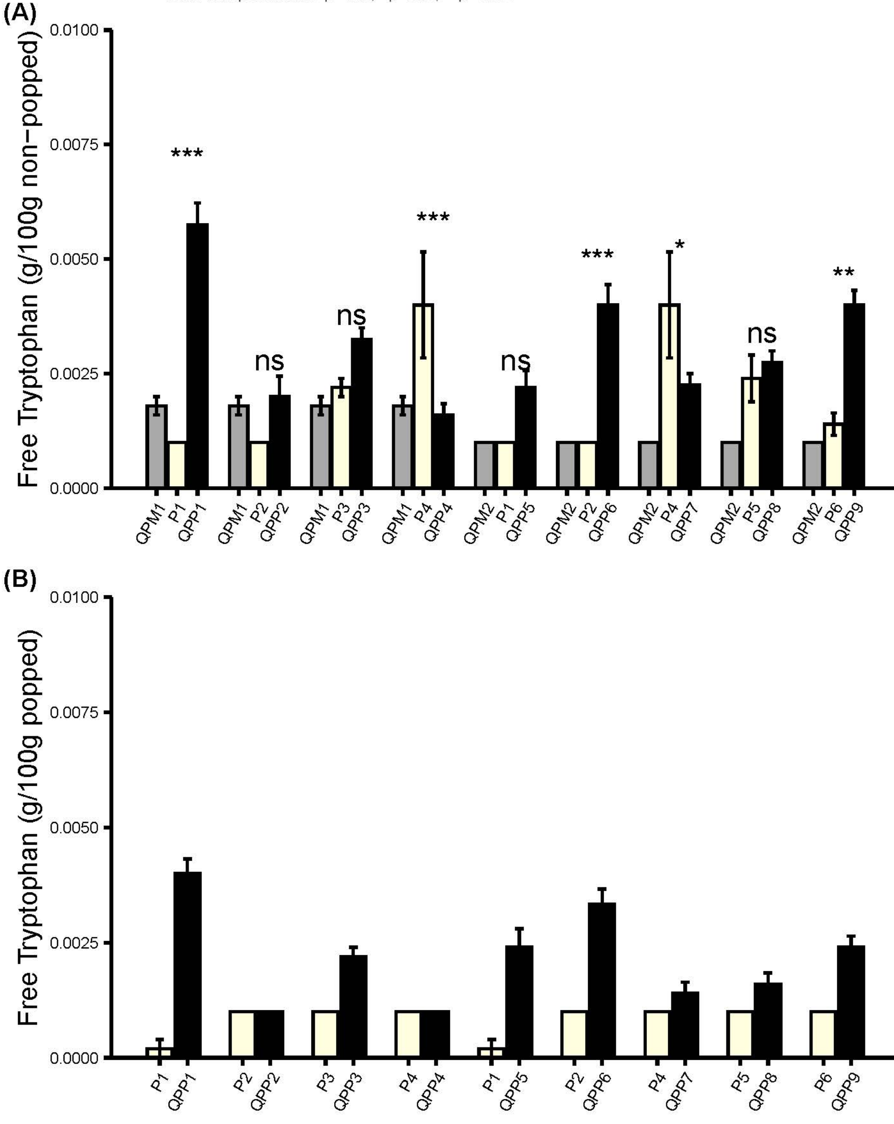

Supplementary Figure S10| Histidine content in QPP inbreds. (A) PBAA in non-popped lines (B) PBAA in popped lines. (C) FAA in non-popped lines (D) FAA in popped lines

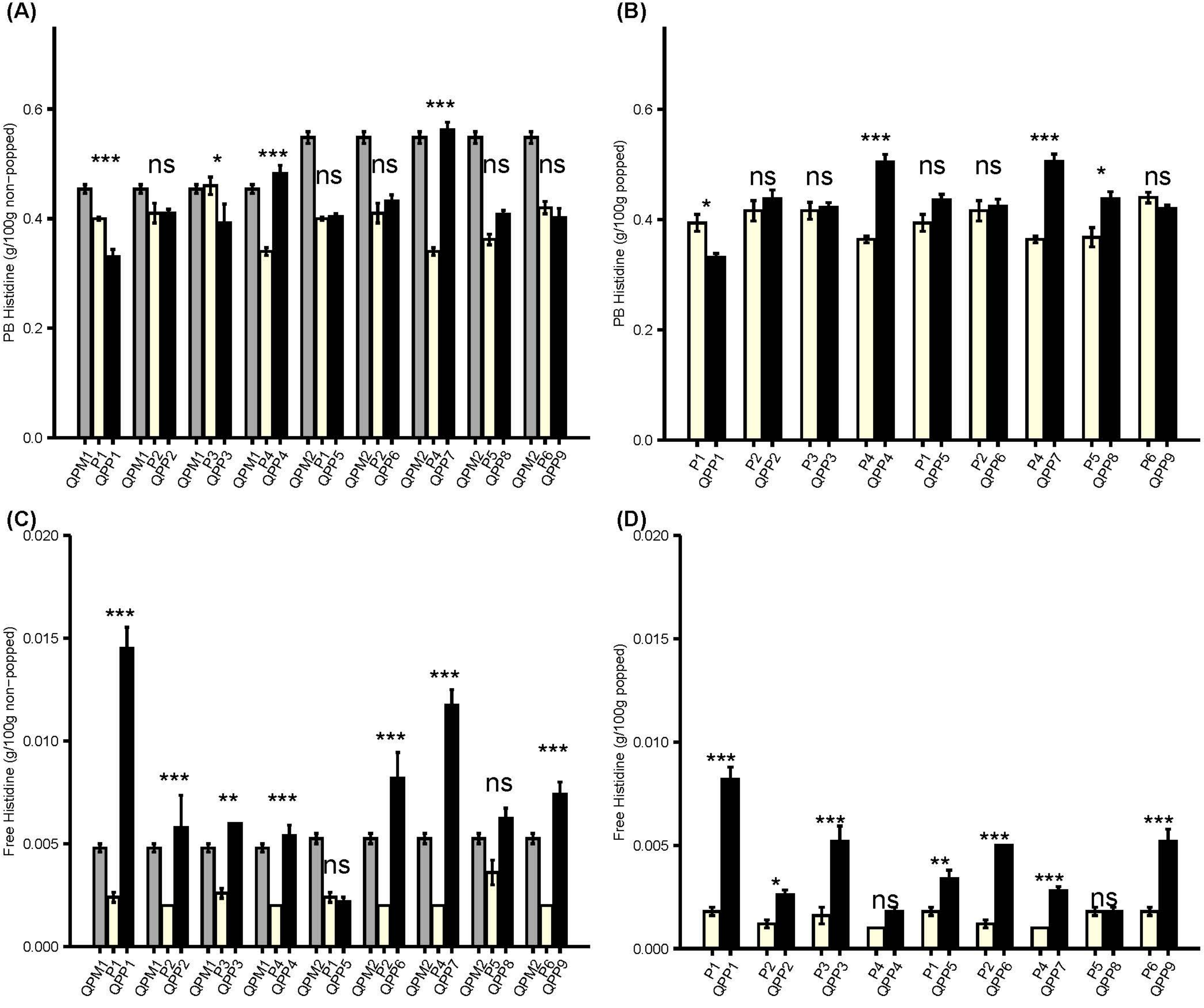

Supplementary Figure S11| Isoleucine content in QPP inbreds. (A) PBAA in non-popped lines (B) PBAA in popped lines. (C) FAA in non-popped lines (D) FAA in popped lines.

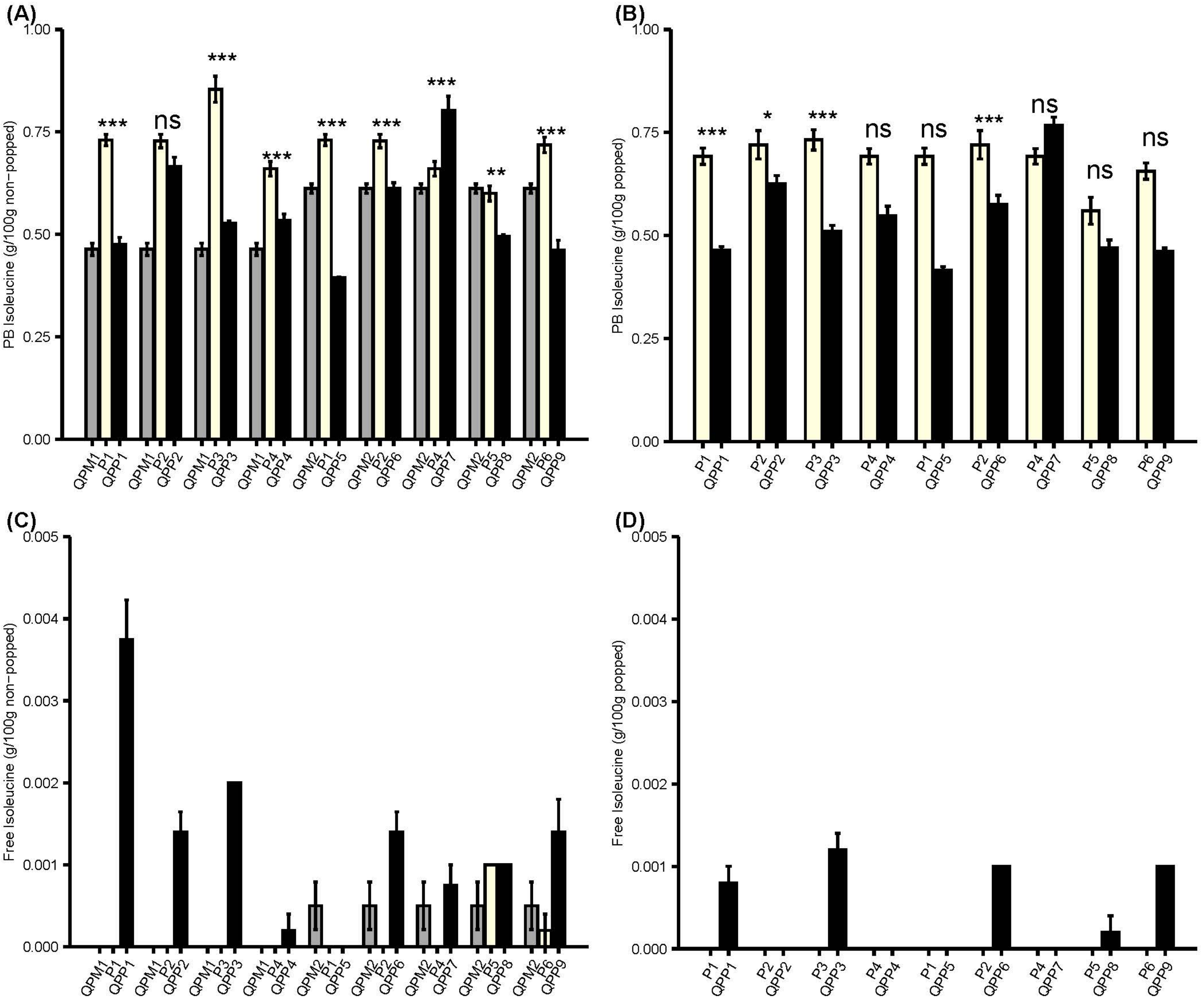

Supplementary Figure S12| Leucine content in QPP inbreds. (A) PBAA in non-popped lines (B) PBAA in popped lines. (C) FAA in non-popped lines (D) FAA in popped lines.

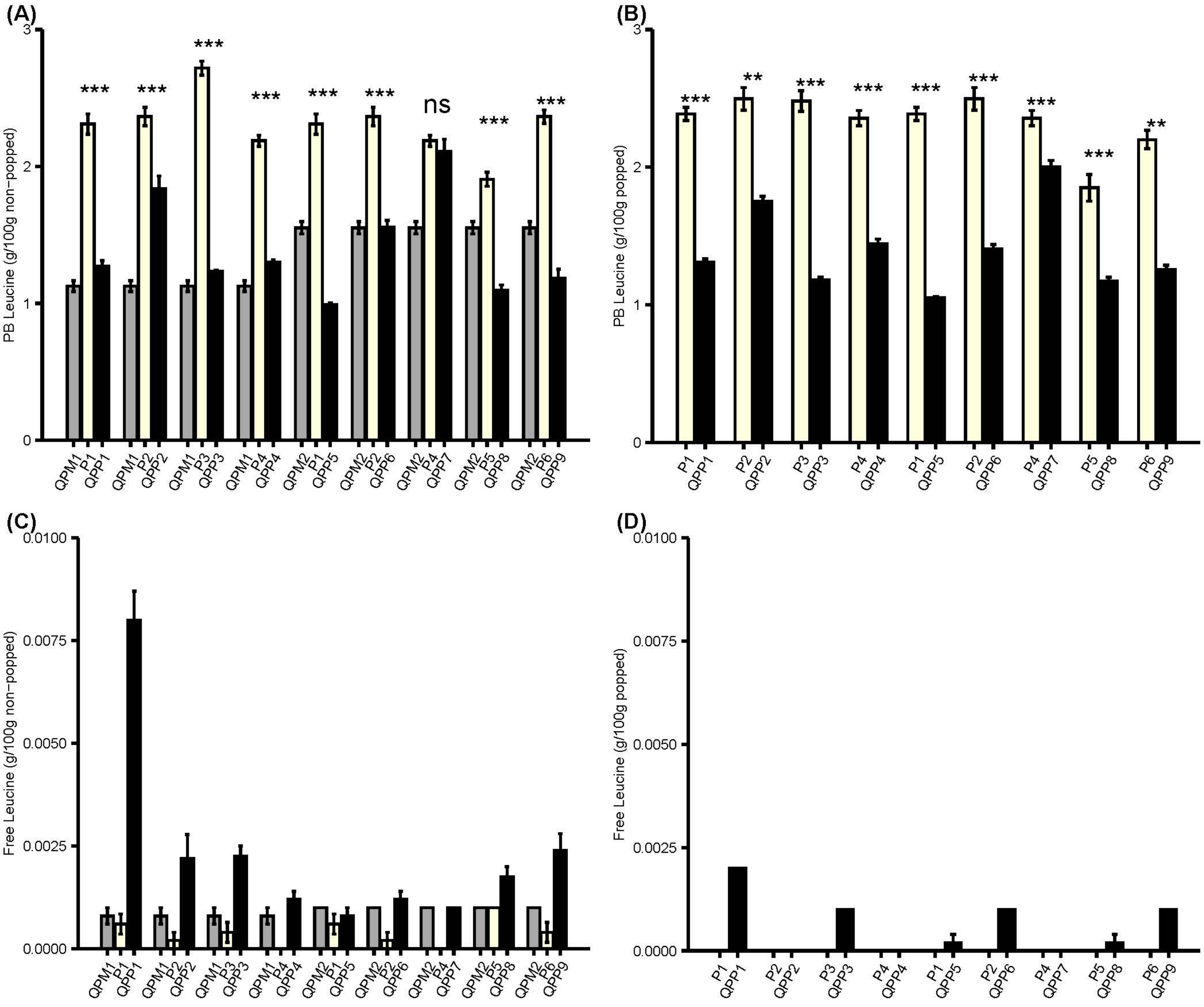

Supplementary Figure S13| Methionine content in QPP inbreds. (A) PBAA in non-popped lines (B) PBAA in popped lines. (C) FAA in non-popped lines (D) FAA in popped lines

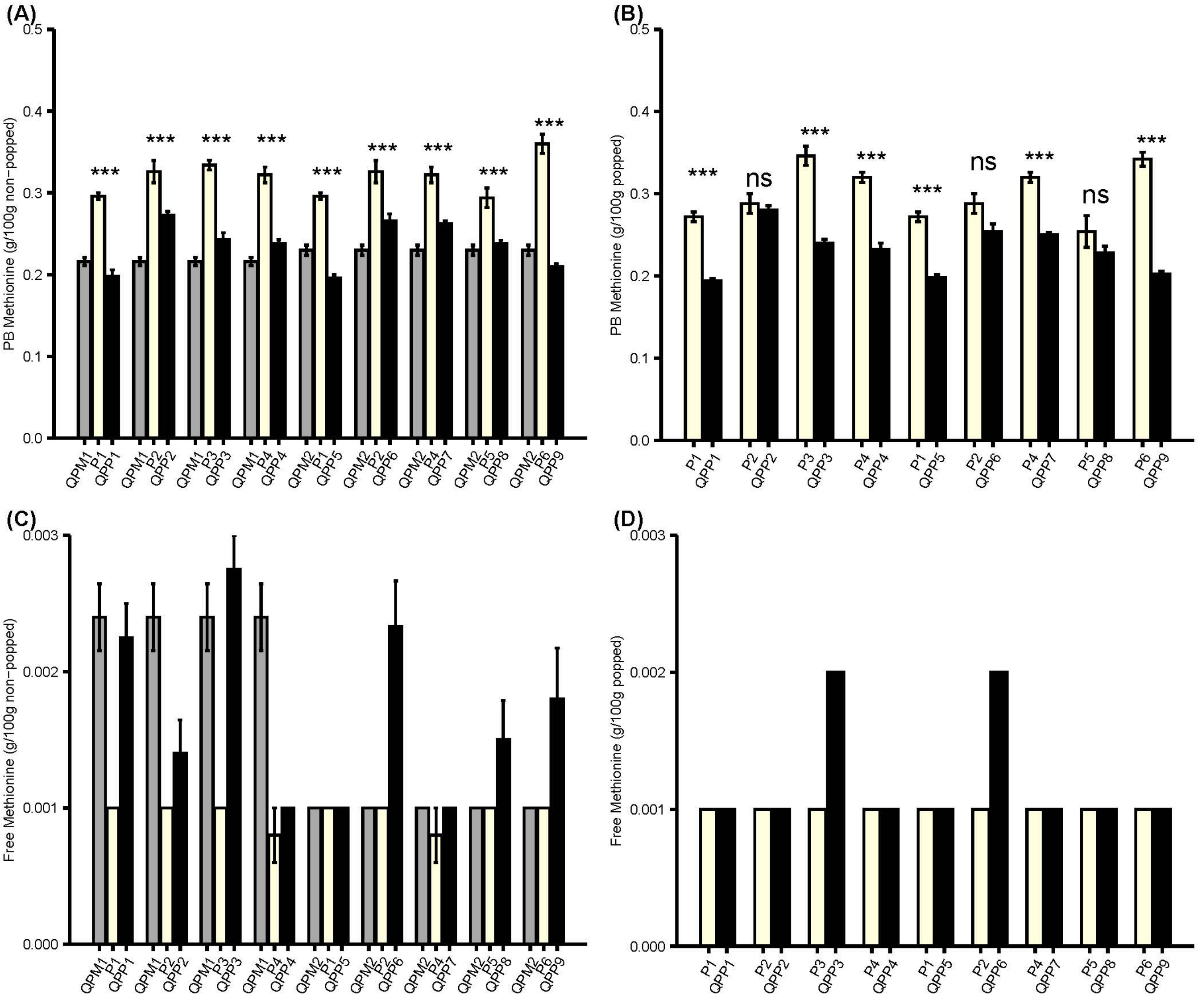

Supplementary Figure S14| Phenylalanine content in QPP inbreds. (A) PBAA in non-popped lines (B) PBAA in popped lines. (C) FAA in non-popped lines (D) FAA in popped lines

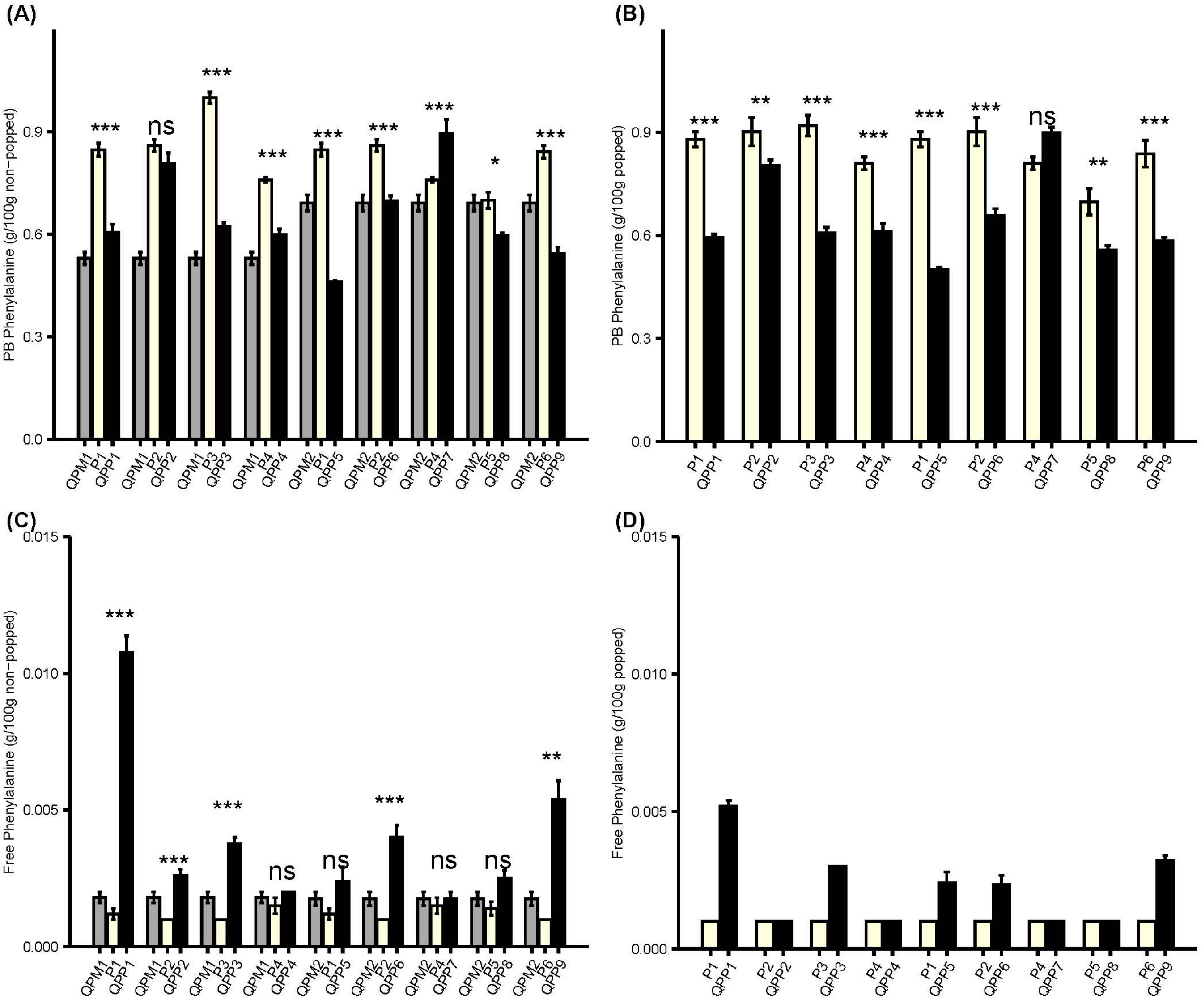

Supplementary Figure S15| Threonine content in QPP inbreds. (A) PBAA in non-popped lines (B) PBAA in popped lines. (C) FAA in non-popped lines (D) FAA in popped lines

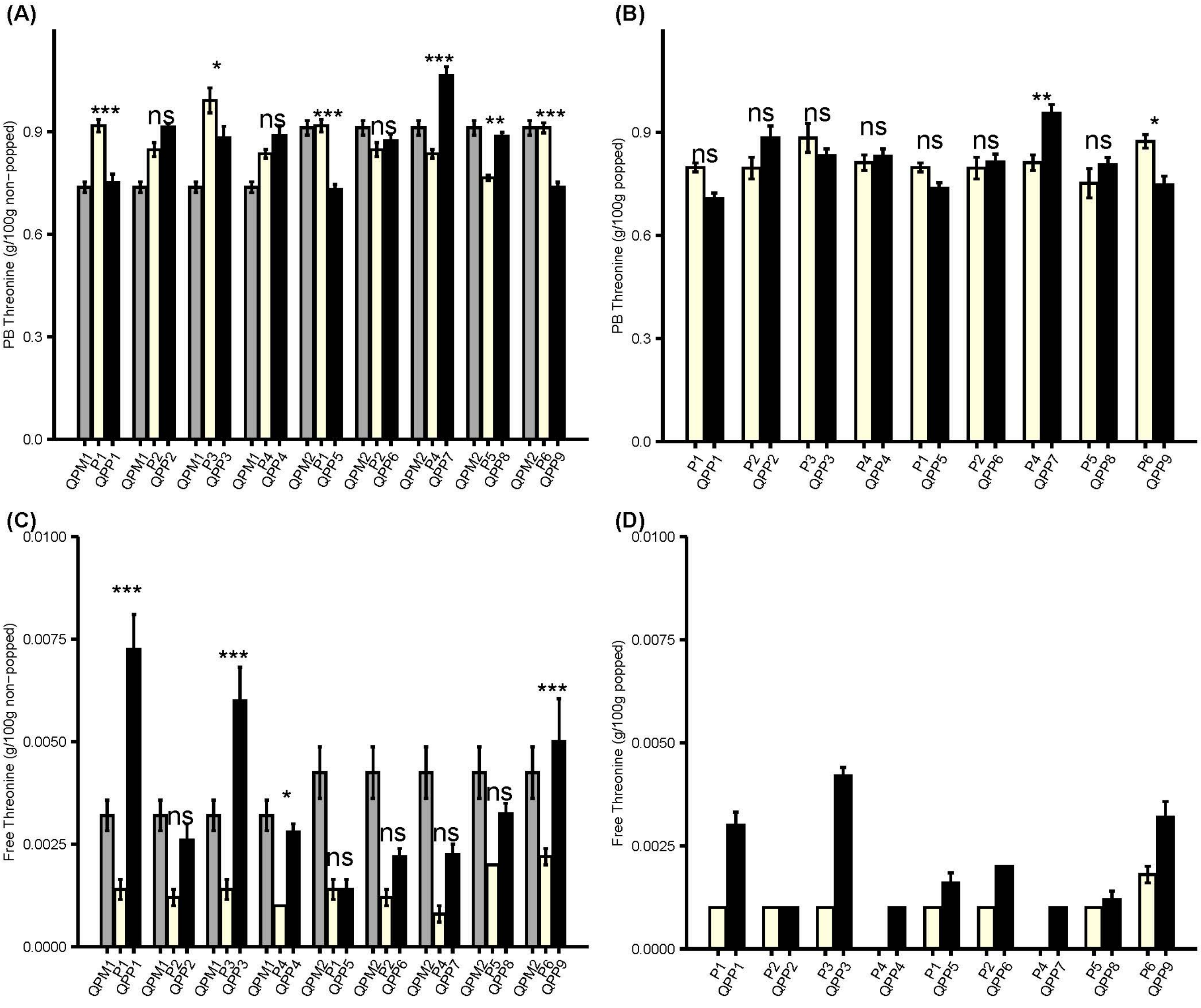

Supplementary Figure S16| Valine content in QPP inbreds. (A) PBAA in non-popped lines (B) PBAA in popped lines. (C) FAA in non-popped lines (D) FAA in popped lines

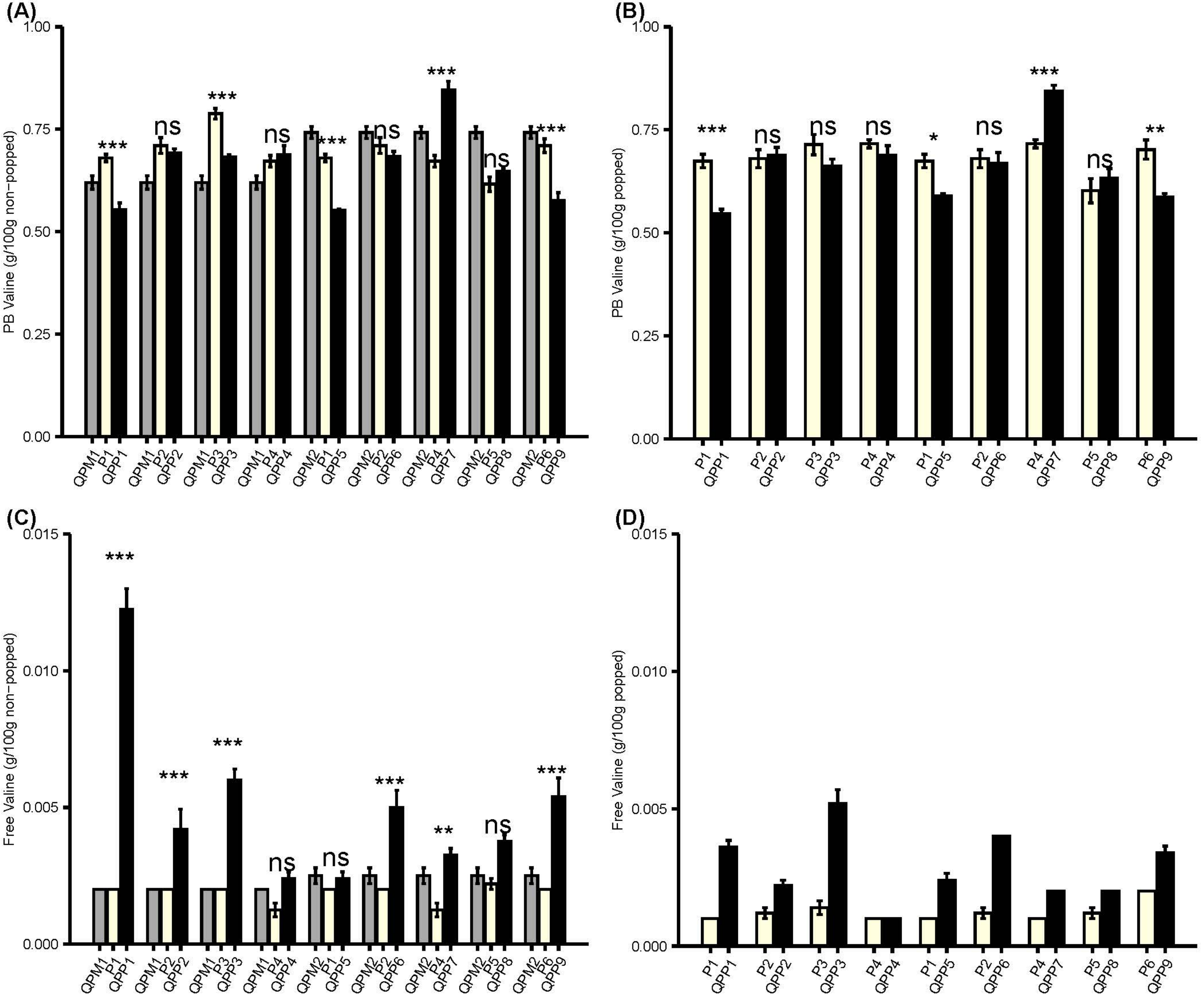

Supplementary Figure S17| Proline content in QPP inbreds. (A) PBAA in non-popped lines (B) PBAA in popped lines. (C) FAA in non-popped lines (D) FAA in popped lines

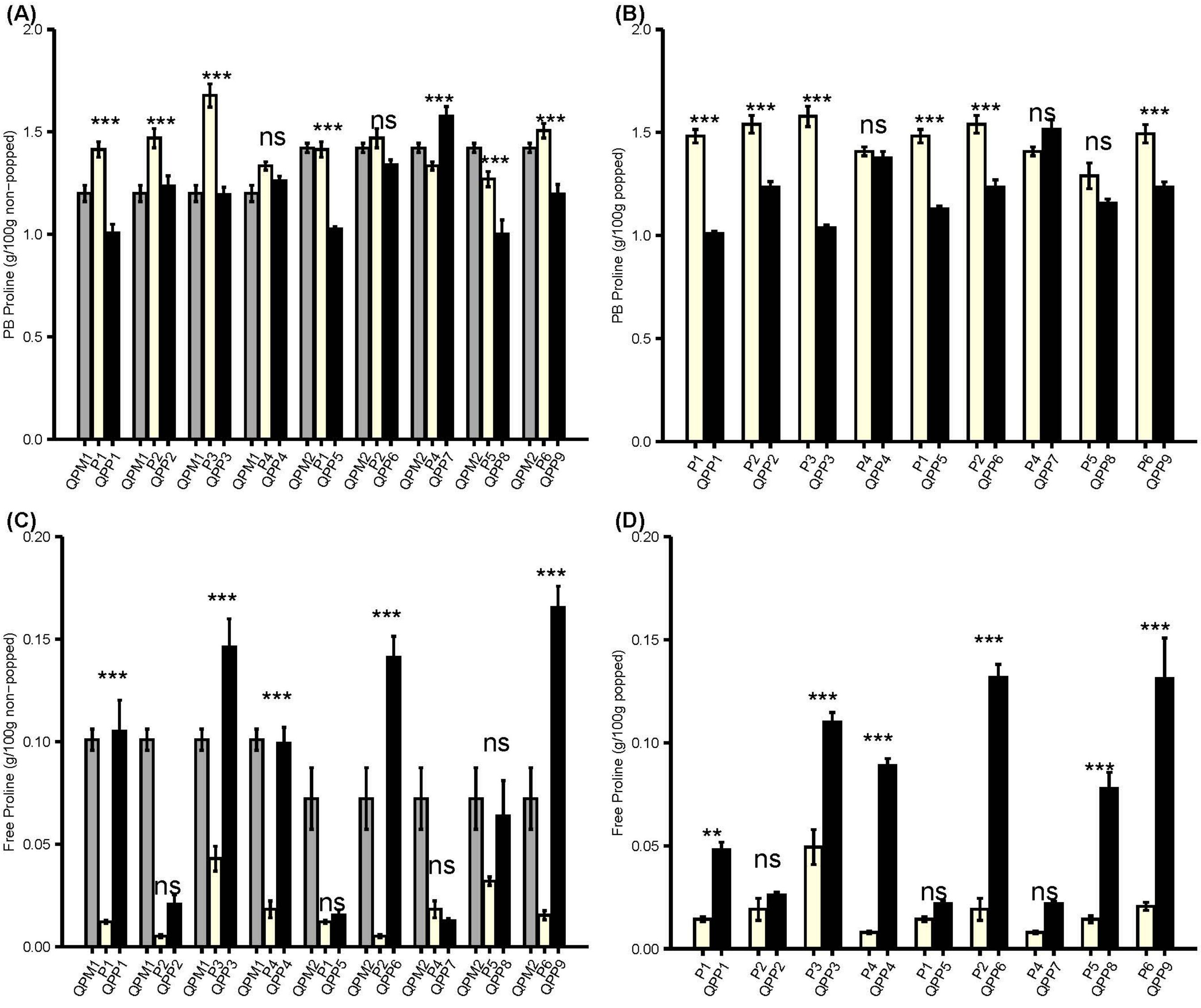

Supplementary Figure S18| Glutamine content in QPP inbreds. (A) PBAA in non-popped lines (B) PBAA in popped lines. (C) FAA in non-popped lines (D) FAA in popped lines

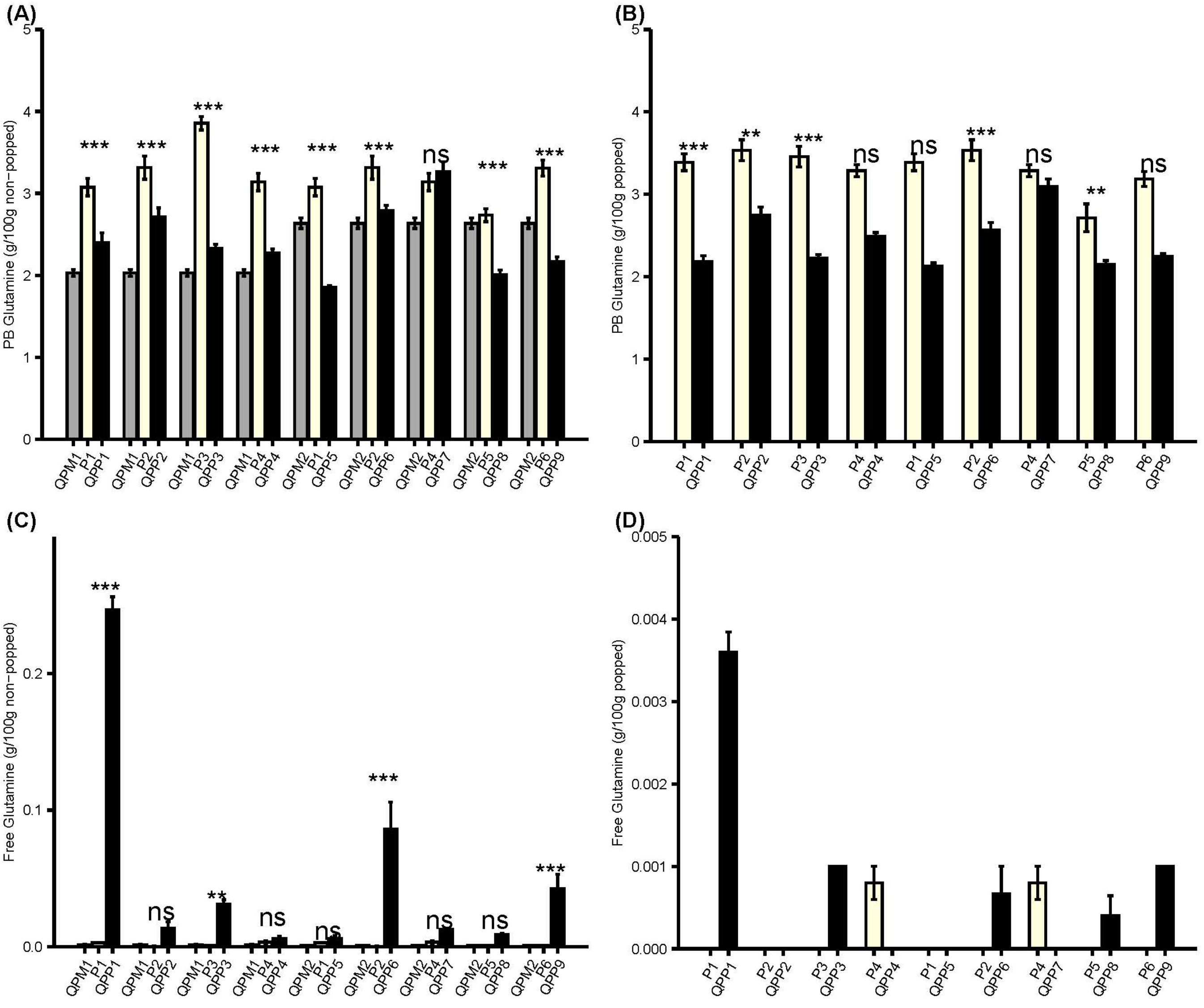

Supplementary Figure S19| Tyrosine content in QPP inbreds. (A) PBAA in non-popped lines (B) PBAA in popped lines. (C) FAA in non-popped lines (D) FAA in popped lines

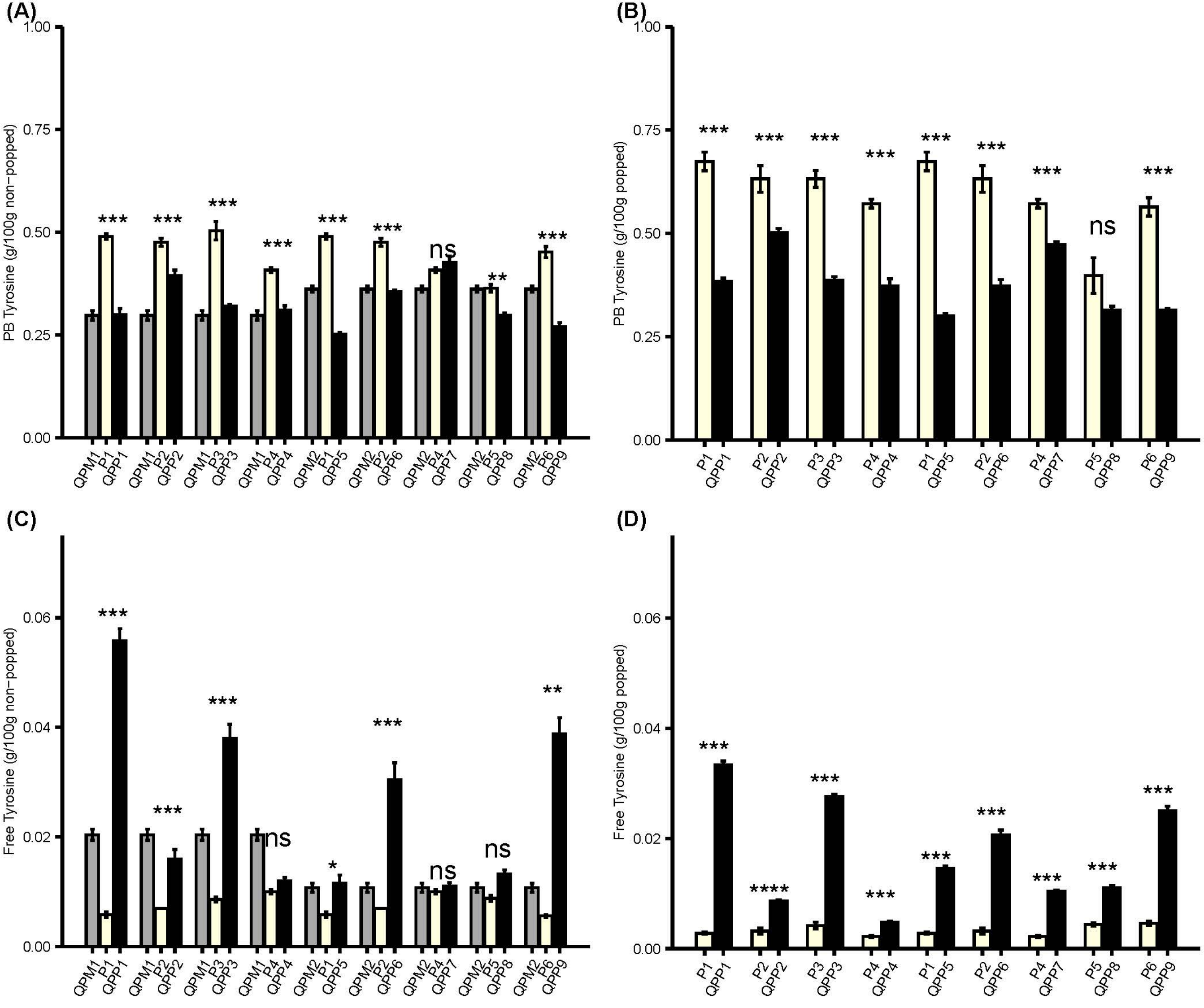

Supplementary Figure S20| Serine content in QPP inbreds. (A) PBAA in non-popped lines (B) PBAA in popped lines. (C) FAA in non-popped lines (D) FAA in popped lines

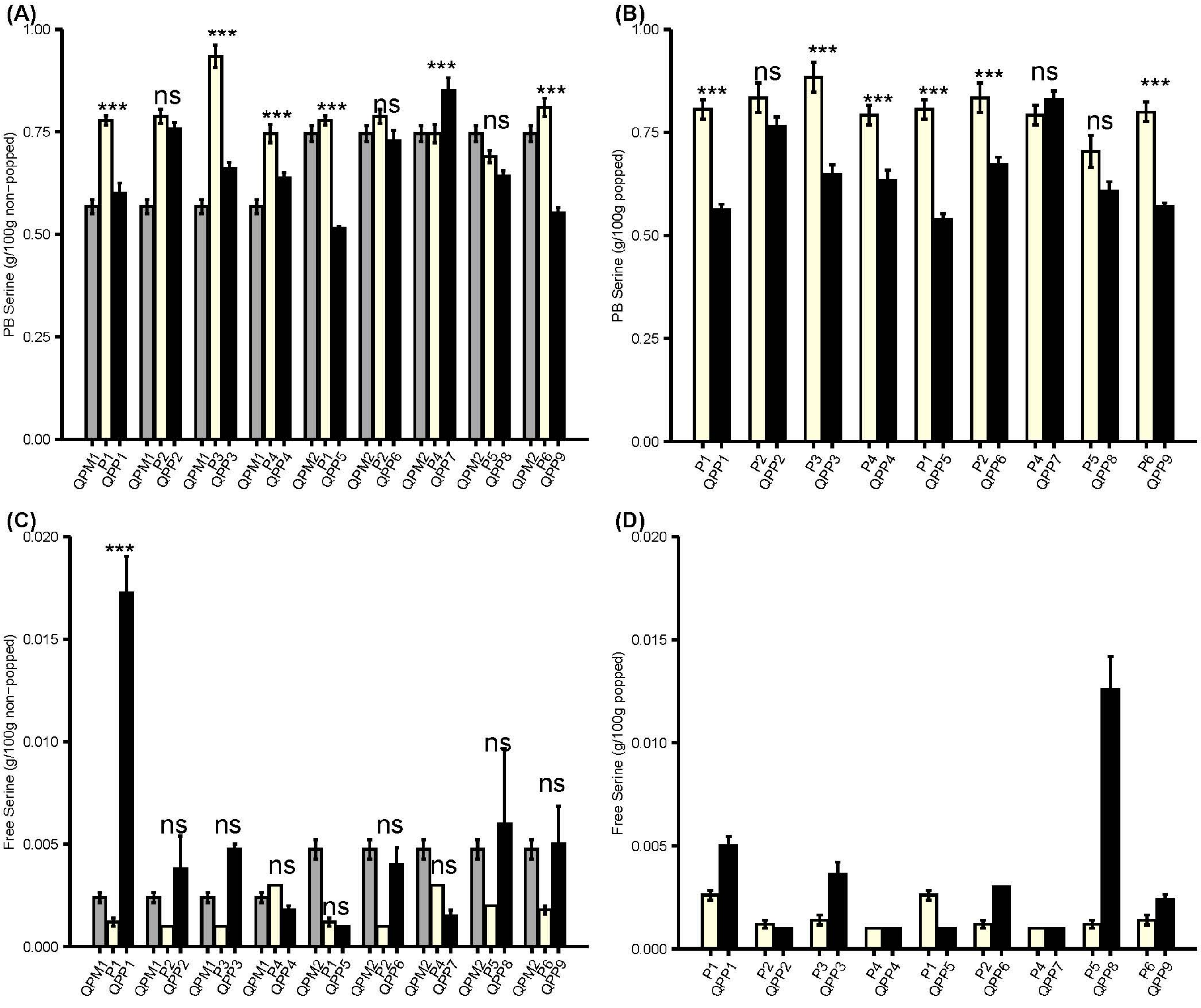

Supplementary Figure S21| Alanine content in QPP inbreds. (A) PBAA in non-popped lines (B) PBAA in popped lines. (C) FAA in non-popped lines (D) FAA in popped lines

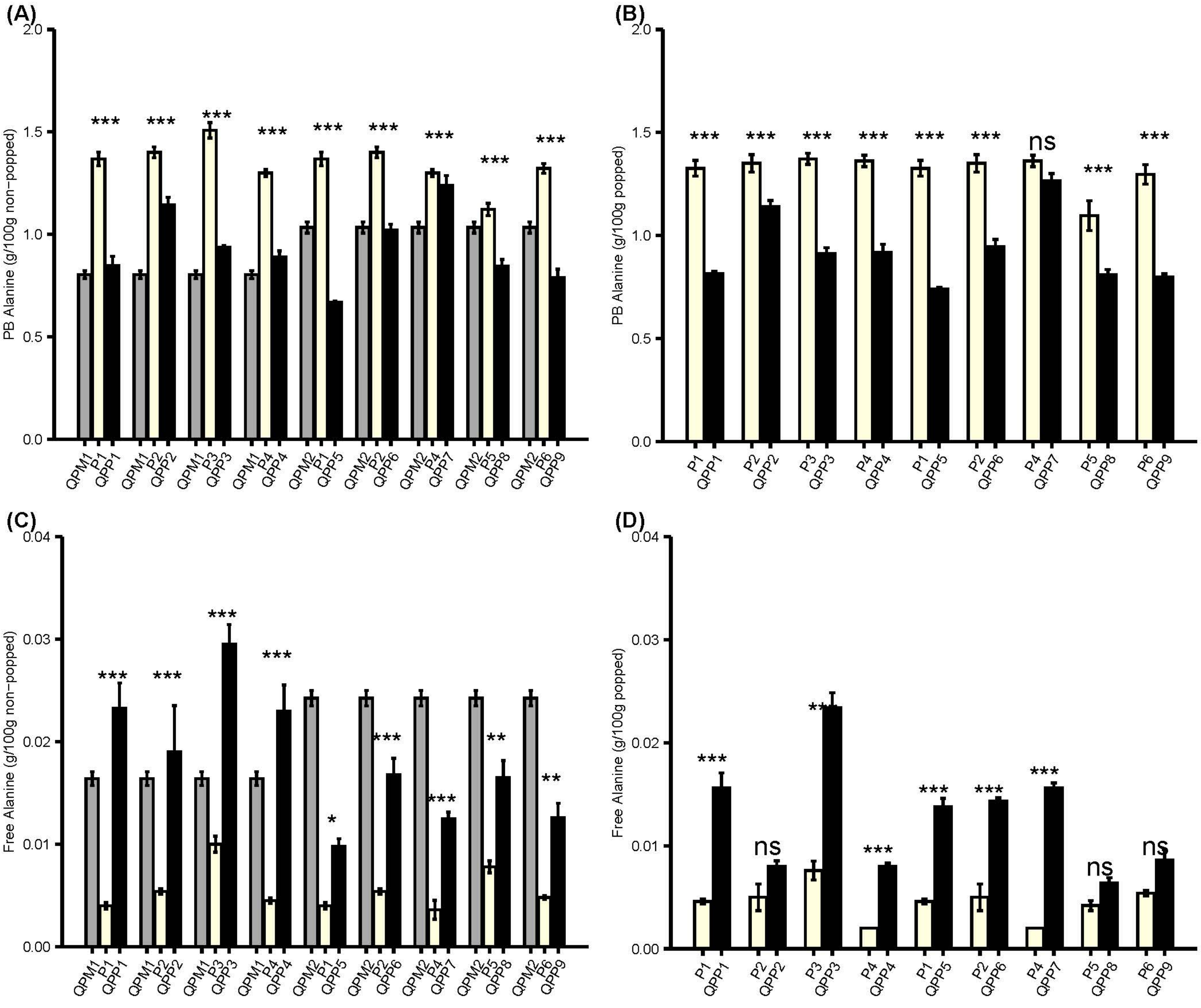

Supplementary Figure S22| Arginine content in QPP inbreds. (A) PBAA in non-popped lines (B) PBAA in popped lines. (C) FAA in non-popped lines (D) FAA in popped lines

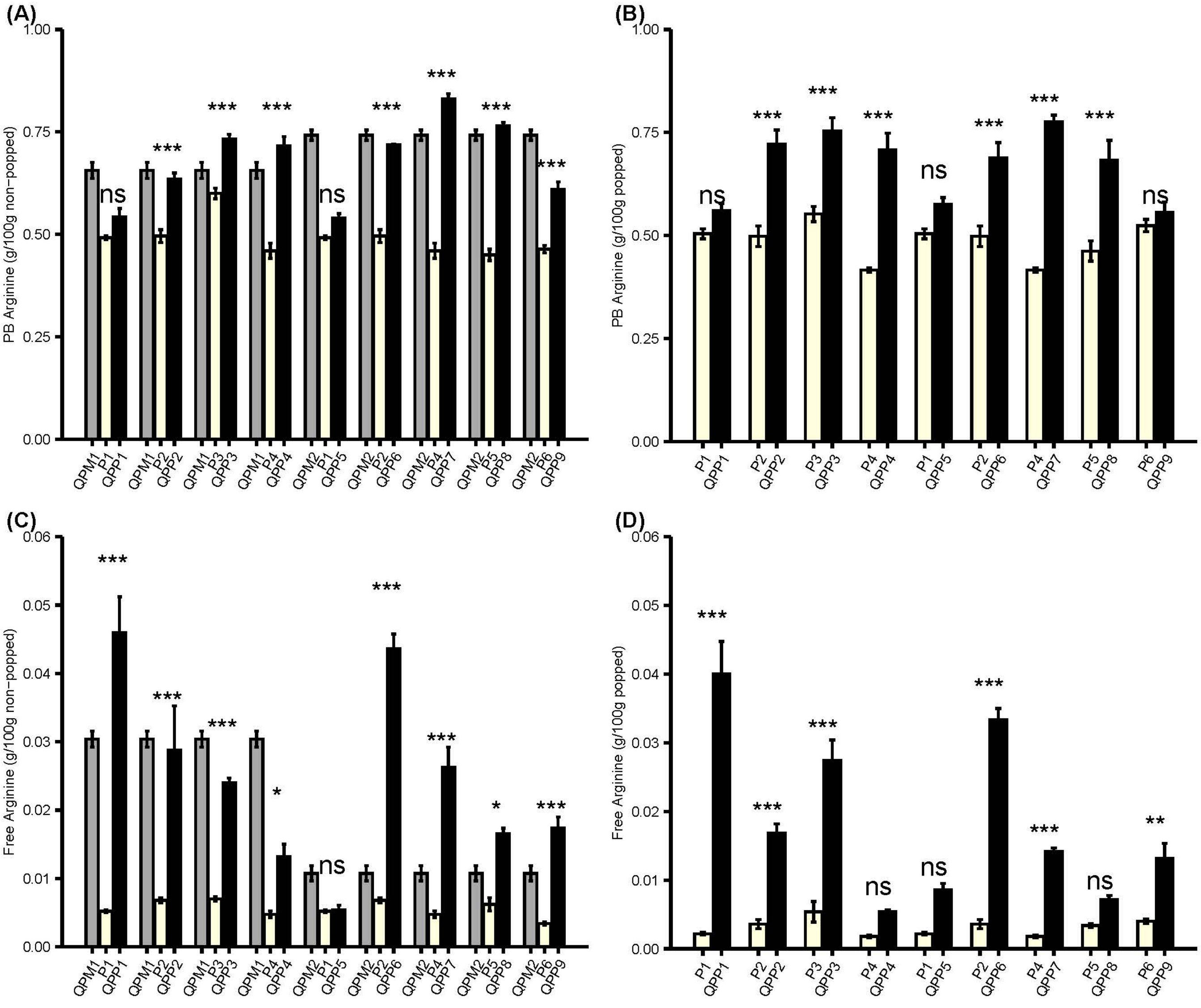

Supplementary Figure S23| Asparagine content in QPP inbreds. (A) PBAA in non-popped lines (B) PBAA in popped lines. (C) FAA in non-popped lines (D) FAA in popped lines

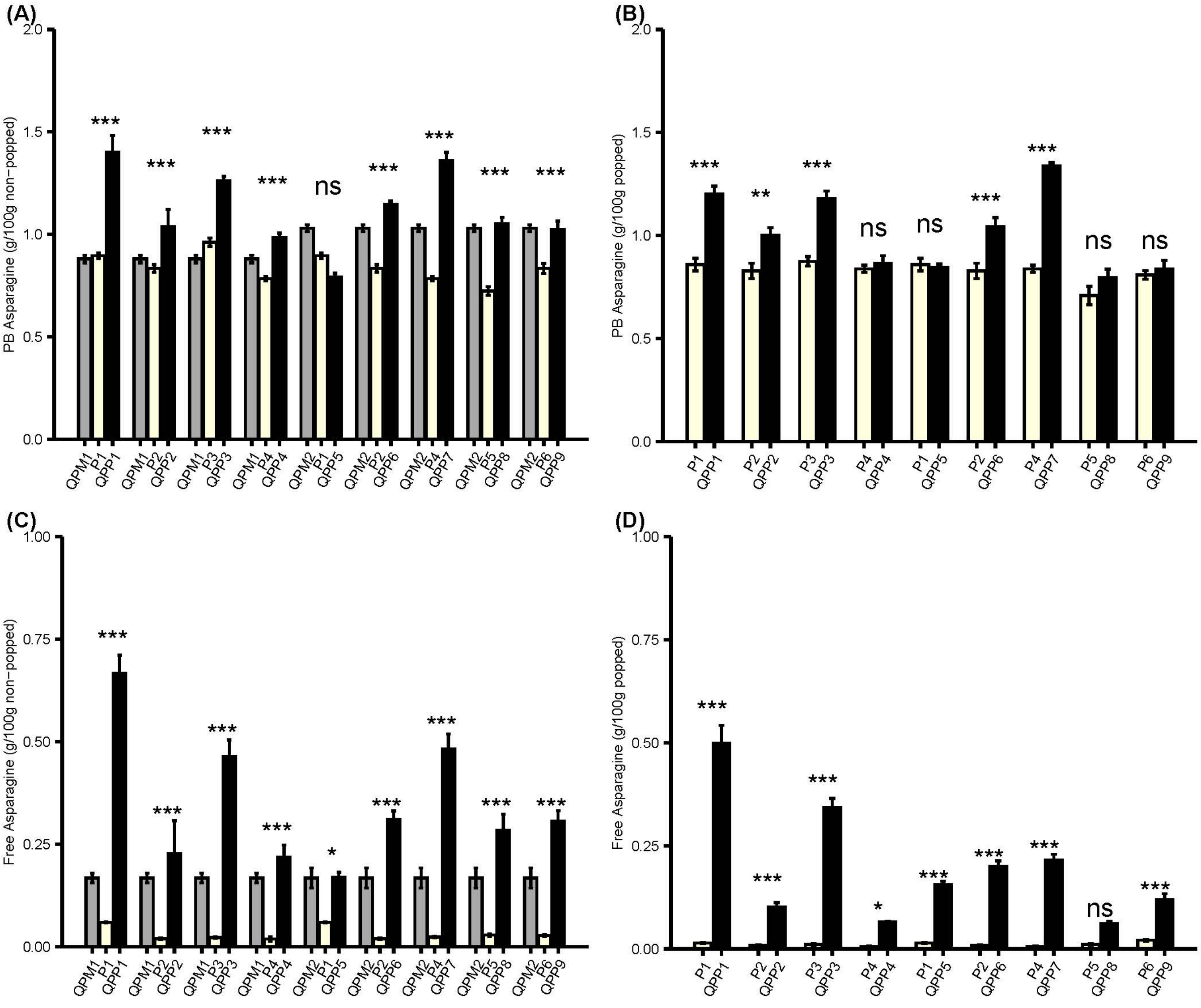

Supplementary Figure S24| Glycine content in QPP inbreds. (A) PBAA in non-popped lines (B) PBAA in popped lines. (C) FAA in non-popped lines (D) FAA in popped lines

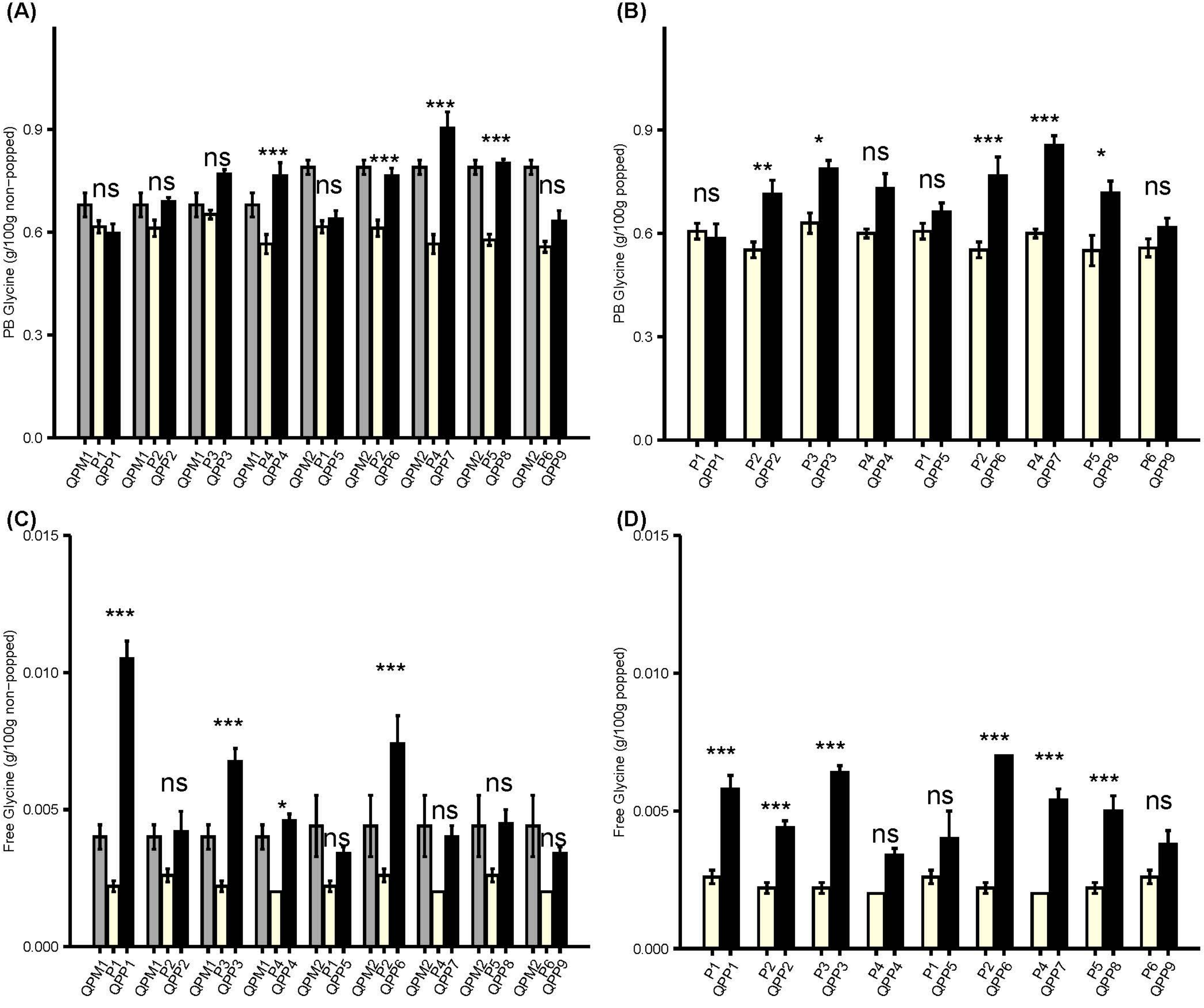

Supplementary Figure S25| Relative flake size of parental colored popcorn and colored QPP

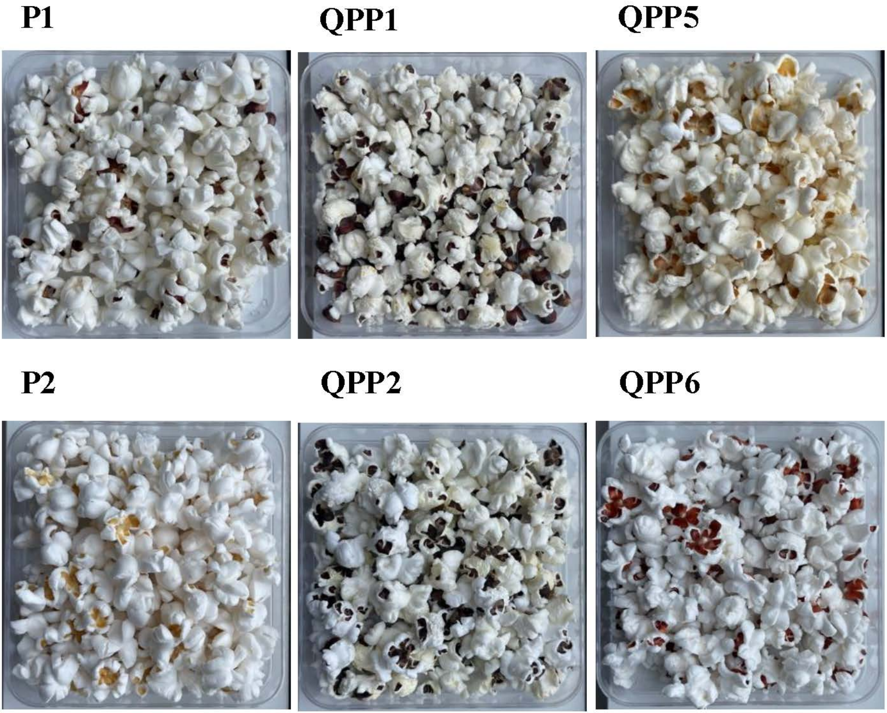

Supplementary Figure S26| Relative flake size of parental colored popcorn and colored QPP.

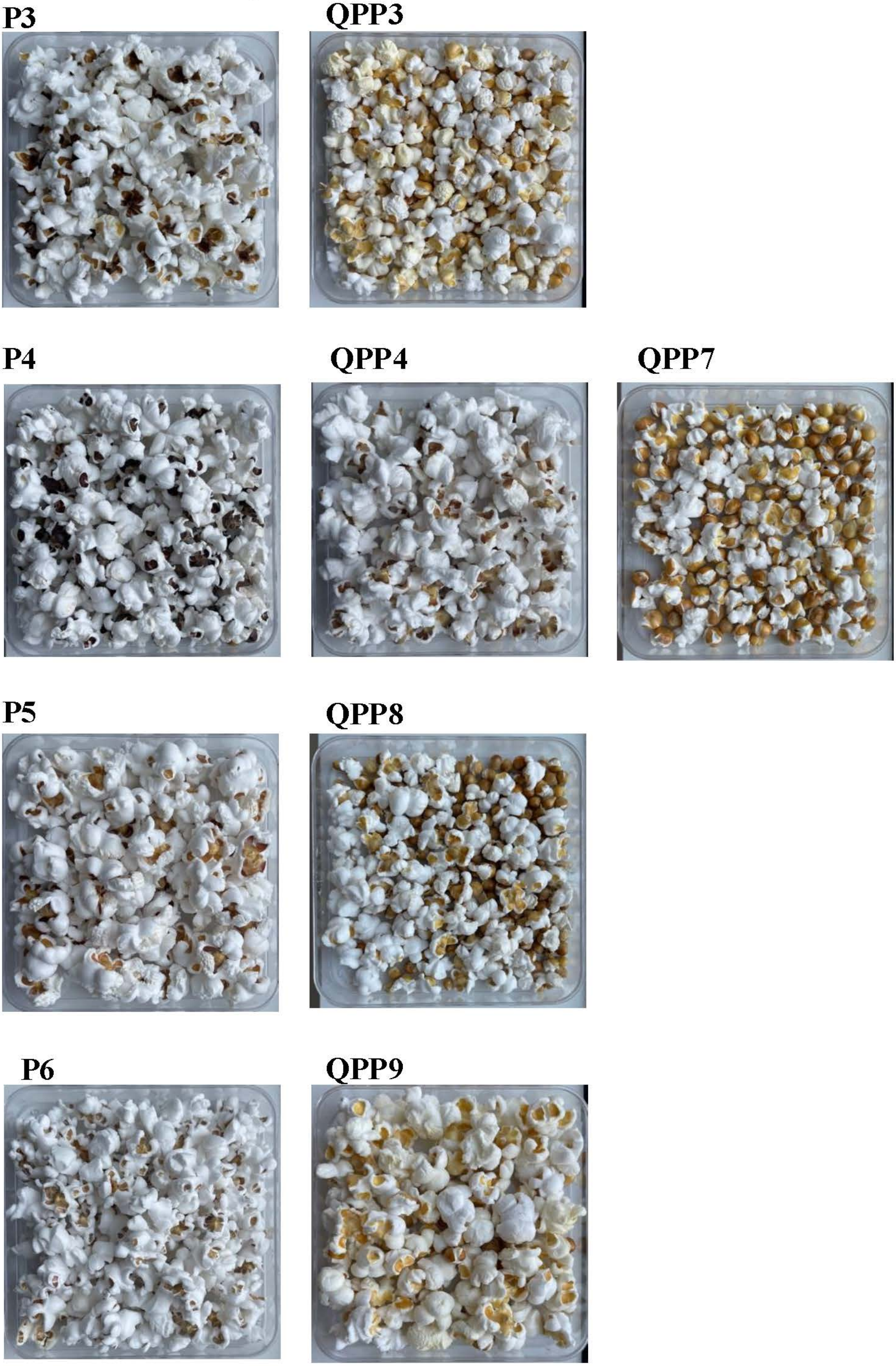

Supplement: Supplementary Figure 1 — Kernel color introgression in F1 QPP lines. [file Image1.pdf]
