# Supplementary material for: Breeding for colored quality protein popcorn with improved amino acid composition
Source: Front Plant Sci. 2026 May 25;17:1844370. doi: 10.3389/fpls.2026.1844370 (PMC13243419; doi:10.3389/fpls.2026.1844370)
Supplement: Supplementary Table 1 — Description of zein levels in colored QPP inbreds. [file Table1.pdf]

Zein levels observed in colored QPP inbreds			
	**19 kDa alpha zeins**	**22 kDa alpha zeins**	**27 kDa gamma zeins**
**QPP1**	low	low	low
**QPP2**	nornal	low	normal
**QPP3**	low	low	normal
**QPP4**	normal	low	high
**QPP5**	low	low	normal
**QPP6**	normal	low	normal
**QPP7**	normal	low	increased
**QPP8**	low	low	low
**QPP9**	normal	low	increased

Supplementary Table S1| Description of zein levels in colored QPP inbreds.

Total protein concentrations (ug/mL)						
	**Rep1**	**Rep2**	**Rep3**	**Rep4**	**Mean**	***sd***
**QPM1**	867	872	803	1152	923	155
**P1**	778	705	919	831	808	90
**QPP1**	1098	975	1112	1090	1069	63
**QPM1**	867	872	803	1152	923	155
**P2**	974	1010	1151	1087	1056	79
**QPP2**	1101	966	1031	880	995	94
**QPM1**	867	872	803	1152	923	155
**P3**	933	1209	1135	1028	1076	121
**QPP3**	1176	942	875	963	989	130
**QPM1**	867	872	803	1152	923	155
**P4**	794	946	1024	819	896	108
**QPP4**	908	828	1086	1054	969	122
**QPM2**	712	922	825	1113	893	170
**P1**	778	705	919	831	808	90
**QPP5**	834	793	813	962	851	76
**QPM2**	712	922	825	1113	893	170
**P2**	974	1010	1151	1087	1056	79
**QPP6**	881	1023	976	894	943	68
**QPM2**	712	922	825	1113	893	170
**P4**	794	946	1024	819	896	108
**QPP7**	858	1022	1146	839	966	145
**QPM2**	712	922	825	1113	893	170
**P5**	832	945	842	733	838	87
**QPP8**	1118	786	806	1114	956	185
**QPM2**	712	922	825	1113	893	170
**P6**	1047	810	1006	852	929	115
**QPP9**	1017	677	712	904	827	161

Supplementary Table S2| Total protein concentrations in QPM, popcorn, and colored QPP inbreds lines

		Ala	Arg	Asx	Glx	Gly	His	Ile	Leu	Lys	Met	Phe	Pro	Ser	Thr	Tyr	Val
	**P1**	1.33	0.50	0.86	3.39	0.61	0.39	0.69	2.38	0.26	0.27	0.88	1.48	0.80	0.80	0.67	0.67
	**P2**	1.35	0.50	0.83	3.53	0.55	0.42	0.72	2.50	0.25	0.29	0.90	1.54	0.83	0.80	0.63	0.68
	**P3**	1.37	0.55	0.87	3.46	0.63	0.42	0.73	2.48	0.28	0.35	0.92	1.58	0.88	0.88	0.63	0.72
	**P4**	1.36	0.42	0.84	3.29	0.60	0.36	0.69	2.36	0.19	0.32	0.81	1.41	0.79	0.81	0.57	0.72
	**P5**	1.09	0.46	0.71	2.71	0.55	0.37	0.56	1.85	0.28	0.25	0.70	1.29	0.70	0.75	0.40	0.60
	**P6**	1.30	0.52	0.81	3.18	0.56	0.44	0.66	2.20	0.32	0.34	0.84	1.49	0.80	0.87	0.57	0.70
**Popped**	**QPP1**	0.81	0.56	1.20	2.18	0.59	0.33	0.46	1.31	0.41	0.19	0.59	1.01	0.56	0.71	0.38	0.55
	**QPP2**	1.14	0.72	1.00	2.75	0.71	0.44	0.63	1.75	0.45	0.28	0.80	1.23	0.76	0.88	0.50	0.69
	**QPP3**	0.91	0.76	1.18	2.22	0.79	0.42	0.51	1.18	0.58	0.24	0.61	1.04	0.65	0.83	0.39	0.66
	**QPP4**	0.92	0.71	0.87	2.49	0.73	0.50	0.55	1.44	0.41	0.23	0.61	1.38	0.63	0.83	0.37	0.69
	**QPP5**	0.74	0.58	0.85	2.13	0.66	0.43	0.41	1.05	0.37	0.20	0.50	1.13	0.54	0.74	0.30	0.59
	**QPP6**	0.95	0.69	1.04	2.56	0.77	0.42	0.58	1.40	0.52	0.25	0.66	1.24	0.67	0.81	0.37	0.67
	**QPP7**	1.26	0.78	1.34	3.10	0.86	0.51	0.77	2.00	0.44	0.25	0.90	1.52	0.83	0.96	0.47	0.84
	**QPP8**	0.81	0.68	0.80	2.15	0.72	0.44	0.47	1.17	0.44	0.23	0.56	1.16	0.61	0.81	0.31	0.63
	**QPP9**	0.80	0.56	0.84	2.24	0.62	0.42	0.47	1.25	0.38	0.20	0.58	1.23	0.57	0.75	0.31	0.59
	**P1**	1.37	0.49	0.90	3.08	0.61	0.40	0.73	2.31	0.41	0.30	0.85	1.41	0.78	0.92	0.49	0.68
	**P2**	1.40	0.50	0.83	3.31	0.61	0.41	0.73	2.36	0.37	0.33	0.86	1.47	0.79	0.85	0.48	0.71
	**P3**	1.51	0.60	0.96	3.85	0.65	0.46	0.85	2.72	0.38	0.33	1.00	1.68	0.94	0.99	0.50	0.79
	**P4**	1.30	0.46	0.78	3.14	0.57	0.34	0.66	2.19	0.34	0.32	0.76	1.33	0.75	0.84	0.41	0.67
	**P5**	1.12	0.45	0.72	2.74	0.58	0.36	0.60	1.91	0.35	0.29	0.70	1.27	0.69	0.77	0.36	0.62
	**P6**	1.32	0.46	0.83	3.31	0.56	0.42	0.72	2.36	0.34	0.36	0.84	1.51	0.81	0.91	0.45	0.71
**Non**	**QPP1**	0.85	0.54	1.40	2.40	0.60	0.33	0.48	1.27	0.49	0.20	0.61	1.01	0.60	0.75	0.30	0.56
**Popped**	**QPP2**	1.14	0.63	1.04	2.71	0.69	0.41	0.67	1.84	0.53	0.27	0.81	1.24	0.76	0.92	0.39	0.69
	**QPP3**	0.94	0.73	1.26	2.33	0.77	0.39	0.53	1.23	0.66	0.24	0.62	1.20	0.66	0.88	0.32	0.68
	**QPP4**	0.89	0.72	0.99	2.27	0.77	0.48	0.53	1.30	0.59	0.24	0.60	1.26	0.64	0.89	0.31	0.69
	**QPP5**	0.67	0.54	0.79	1.86	0.64	0.40	0.39	0.99	0.46	0.20	0.46	1.03	0.51	0.73	0.25	0.55
	**QPP6**	1.02	0.72	1.15	2.79	0.77	0.43	0.61	1.56	0.59	0.27	0.70	1.34	0.73	0.88	0.36	0.69
	**QPP7**	1.24	0.83	1.36	3.27	0.90	0.56	0.80	2.11	0.63	0.26	0.90	1.58	0.85	1.07	0.43	0.84
	**QPP8**	0.84	0.77	1.05	2.01	0.80	0.41	0.50	1.10	0.64	0.24	0.60	1.00	0.64	0.89	0.30	0.65
	**QPP9**	0.79	0.61	1.02	2.17	0.63	0.40	0.46	1.18	0.48	0.21	0.55	1.19	0.55	0.74	0.27	0.58

Supplementary Table S3| Mean PBAA values of parental popcorns and colored QPP inbreds.

	Ala	Arg	Asx	Glx	Gly	His	Ile	Leu	Lys	Met	Phe	Pro	Ser	Thr	Tyr	Val
**non-popped P1/popped P1**	1.03	0.97	1.045	0.91	1.01	1.013	1.05	0.969	1.591	1.086	0.96	0.954	0.97	1.15	0.728	1.01
**non-popped P2/popped P2**	1.04	1	1.007	0.94	1.11	0.982	1.01	0.947	1.455	1.144	0.95	0.955	0.95	1.065	0.756	1.04
**non-popped P3/popped P3**	1.1	1.08	1.099	1.12	1.04	1.104	1.17	1.096	1.351	0.959	1.09	1.064	1.06	1.122	0.797	1.1
**non-popped P4/popped P4**	0.96	1.11	0.938	0.95	0.94	0.936	0.96	0.928	1.747	0.997	0.94	0.949	0.94	1.028	0.713	0.94
**non-popped P5/popped P5**	1.03	0.97	1.02	1.01	1.05	0.983	1.07	1.031	1.221	1.152	1	0.983	0.98	1.021	0.919	1.02
**non-popped P6/popped P6**	1.02	0.89	1.031	1.04	0.99	0.949	1.1	1.075	1.087	1.047	1.01	1.009	1.01	1.044	0.796	1.01
**non-popped QPP1/popped QPP1**	1.04	0.97	1.167	1.1	1.02	0.992	1.03	0.972	1.195	1.02	1.02	0.999	1.06	1.064	0.782	1.02
**non-popped QPP2/popped QPP2**	1	0.88	1.036	0.99	0.97	0.933	1.06	1.05	1.176	0.973	1.01	1.003	0.99	1.038	0.787	1.01
**non-popped QPP3/popped QPP3**	1.03	0.97	1.07	1.05	0.97	0.941	1.04	1.044	1.141	1.015	1.02	1.151	1.02	1.061	0.833	1.03
**non-popped QPP4/popped QPP4**	0.97	1.01	1.139	0.91	1.05	0.95	0.98	0.901	1.446	1.02	0.98	0.916	1.01	1.072	0.835	1
**non-popped QPP5/popped QPP5**	0.91	0.94	0.938	0.87	0.97	0.931	0.95	0.941	1.233	1	0.92	0.911	0.95	0.993	0.844	0.93
**non-popped QPP6/popped QPP6**	1.08	1.04	1.103	1.09	1	1.021	1.06	1.11	1.13	1.055	1.06	1.085	1.08	1.077	0.955	1.03
**non-popped QPP7/popped QPP7**	0.98	1.07	1.016	1.06	1.06	1.107	1.04	1.052	1.44	1.064	1	1.042	1.03	1.118	0.903	1
**non-popped QPP8/popped QPP8**	1.04	1.12	1.32	0.94	1.11	0.936	1.05	0.935	1.455	1.042	1.07	0.866	1.06	1.101	0.949	1.03
**non-popped QPP9/popped QPP9**	0.98	1.1	1.225	0.97	1.02	0.954	1	0.942	1.263	1.037	0.93	0.967	0.97	0.987	0.863	0.98

Supplementary Table S4| Non-popped/popped ratio values of PBAA fractions in parental popcorn and colored QPP inbreds.

		Ala	Arg	Asx	Glx	Gly	His	Ile	Leu	Lys	Met	Phe	Pro	Ser	Thr	Tyr	Val
	**QPP1/P1**	0.61	1.11	1.40	0.64	0.97	0.84	0.67	0.55	1.59	0.71	0.68	0.68	0.70	0.89	0.57	0.81
	**QPP2/P2**	0.85	1.45	1.21	0.78	1.29	1.05	0.87	0.70	1.78	0.97	0.89	0.80	0.92	1.11	0.79	1.01
	**QPP3/P3**	0.66	1.37	1.35	0.64	1.25	1.01	0.70	0.48	2.08	0.69	0.66	0.66	0.73	0.94	0.61	0.93
	**QPP4/P4**	0.67	1.70	1.04	0.76	1.22	1.39	0.79	0.61	2.11	0.73	0.76	0.98	0.80	1.02	0.65	0.96
**Popped**	**QPP5/P1**	0.56	1.14	0.99	0.63	1.09	1.10	0.60	0.44	1.44	0.72	0.57	0.76	0.67	0.92	0.45	0.88
	**QPP6/P2**	0.70	1.38	1.26	0.73	1.39	1.02	0.80	0.56	2.05	0.88	0.73	0.80	0.81	1.02	0.59	0.98
	**QPP7/P4**	0.93	1.86	1.60	0.94	1.43	1.40	1.11	0.85	2.27	0.77	1.11	1.08	1.05	1.17	0.83	1.18
	**QPP8/P5**	0.74	1.48	1.12	0.79	1.31	1.18	0.84	0.63	1.54	0.89	0.80	0.90	0.86	1.07	0.79	1.05
	**QPP9/P6**	0.62	1.06	1.04	0.71	1.10	0.95	0.71	0.57	1.20	0.60	0.70	0.83	0.71	0.86	0.56	0.84
	**QPP1/P1**	0.62	1.11	1.56	0.78	0.97	0.83	0.65	0.55	1.19	0.66	0.72	0.71	0.77	0.82	0.61	0.82
	**QPP2/P2**	0.82	1.28	1.24	0.82	1.12	1.00	0.92	0.78	1.44	0.83	0.94	0.84	0.96	1.08	0.83	0.98
	**QPP3/P3**	0.62	1.23	1.31	0.60	1.18	0.86	0.62	0.45	1.76	0.73	0.62	0.71	0.70	0.89	0.64	0.87
	**QPP4/P4**	0.68	1.56	1.26	0.72	1.36	1.41	0.81	0.59	1.75	0.74	0.79	0.95	0.85	1.06	0.76	1.02
**Non**	**QPP5/P1**	0.49	1.10	0.88	0.60	1.05	1.01	0.54	0.43	1.12	0.67	0.55	0.73	0.66	0.80	0.52	0.81
**popped**	**QPP6/P2**	0.73	1.44	1.38	0.84	1.25	1.06	0.84	0.66	1.59	0.82	0.81	0.91	0.92	1.03	0.74	0.97
	**QPP7/P4**	0.95	1.80	1.73	1.04	1.60	1.65	1.21	0.96	1.87	0.82	1.18	1.18	1.15	1.28	1.05	1.26
	**QPP8/P5**	0.75	1.70	1.46	0.73	1.39	1.13	0.83	0.57	1.84	0.81	0.85	0.79	0.93	1.16	0.81	1.05
	**QPP9/P6**	0.60	1.31	1.23	0.66	1.14	0.96	0.65	0.50	1.39	0.59	0.65	0.79	0.69	0.81	0.60	0.81

Supplementary Table S5| QPP/P ratios of PBAA in popped and non-popped lines.

Supplementary Table S6| Mean FAA values of parental popcorns and colored QPP inbreds

		Ala	Arg	Asn	Asp	Gln	Glu	Gly	His	Ile	Leu	Lys	Met	Phe	Pro	Ser	Trp	Thr	Tyr	Val	Cys
	**P1**	0.004	0.003	0.014	0.012	0.000	0.004	0.003	0.002	0.000	0.000	0.002	0.001	0.001	0.014	0.002	0.000	0.001	0.002	0.001	0.000
	**P2**	0.005	0.004	0.007	0.009	0.000	0.004	0.002	0.001	0.000	0.000	0.002	0.001	0.001	0.019	0.001	0.001	0.001	0.003	0.001	0.000
	**P3**	0.007	0.005	0.011	0.011	0.000	0.005	0.002	0.002	0.000	0.000	0.003	0.001	0.001	0.050	0.001	0.001	0.001	0.004	0.001	0.000
	**P4**	0.002	0.002	0.006	0.003	0.001	0.003	0.002	0.001	0.000	0.000	0.002	0.001	0.001	0.008	0.001	0.001	0.000	0.002	0.001	0.000
	**P5**	0.004	0.003	0.011	0.013	0.000	0.006	0.002	0.002	0.000	0.000	0.002	0.001	0.001	0.014	0.001	0.001	0.001	0.004	0.001	0.000
	**P6**	0.005	0.004	0.021	0.015	0.000	0.006	0.003	0.002	0.000	0.000	0.002	0.001	0.001	0.021	0.001	0.001	0.002	0.004	0.002	0.000
**Popped**	**QPP1**	0.016	0.040	0.499	0.102	0.004	0.038	0.006	0.008	0.001	0.002	0.019	0.001	0.005	0.048	0.005	0.004	0.003	0.033	0.004	0.000
	**QPP2**	0.008	0.017	0.102	0.044	0.000	0.004	0.004	0.003	0.000	0.000	0.010	0.001	0.001	0.026	0.001	0.001	0.001	0.008	0.002	0.000
	**QPP3**	0.023	0.027	0.344	0.122	0.001	0.046	0.006	0.005	0.001	0.001	0.012	0.002	0.003	0.110	0.003	0.002	0.004	0.028	0.005	0.000
	**QPP4**	0.008	0.006	0.065	0.037	0.000	0.011	0.004	0.002	0.000	0.000	0.003	0.001	0.001	0.089	0.001	0.001	0.001	0.005	0.001	0.000
	**QPP5**	0.014	0.009	0.156	0.076	0.000	0.034	0.004	0.003	0.000	0.001	0.007	0.001	0.003	0.022	0.001	0.002	0.001	0.015	0.003	0.000
	**QPP6**	0.014	0.033	0.201	0.078	0.001	0.034	0.007	0.005	0.001	0.001	0.023	0.002	0.002	0.132	0.003	0.003	0.002	0.021	0.004	0.000
	**QPP7**	0.016	0.014	0.215	0.088	0.000	0.007	0.005	0.003	0.000	0.000	0.006	0.001	0.001	0.022	0.001	0.002	0.001	0.010	0.002	0.000
	**QPP8**	0.006	0.007	0.061	0.054	0.000	0.013	0.005	0.002	0.001	0.001	0.004	0.001	0.001	0.078	0.012	0.002	0.001	0.011	0.002	0.000
	**QPP9**	0.009	0.013	0.120	0.102	0.001	0.026	0.004	0.005	0.001	0.001	0.008	0.001	0.003	0.131	0.002	0.002	0.003	0.025	0.003	0.000
	**P1**	0.004	0.005	0.060	0.010	0.003	0.021	0.002	0.002	0.000	0.001	0.004	0.001	0.001	0.012	0.001	0.001	0.001	0.006	0.002	0.000
	**P2**	0.005	0.007	0.020	0.008	0.000	0.014	0.003	0.002	0.000	0.001	0.004	0.001	0.001	0.005	0.001	0.001	0.001	0.007	0.002	0.000
	**P3**	0.010	0.007	0.022	0.015	0.001	0.009	0.002	0.003	0.000	0.001	0.004	0.001	0.001	0.043	0.001	0.002	0.001	0.008	0.002	0.000
	**P4**	0.004	0.005	0.024	0.007	0.004	0.020	0.002	0.002	0.000	0.000	0.004	0.001	0.001	0.018	0.003	0.004	0.001	0.010	0.001	0.000
	**P5**	0.008	0.006	0.028	0.020	0.001	0.015	0.003	0.003	0.001	0.001	0.004	0.001	0.001	0.032	0.002	0.002	0.002	0.009	0.002	0.000
	**P6**	0.005	0.003	0.027	0.014	0.001	0.012	0.002	0.002	0.001	0.001	0.003	0.001	0.001	0.015	0.002	0.001	0.002	0.006	0.002	0.000
**Non**	**QPP1**	0.023	0.046	0.667	0.118	0.247	0.140	0.010	0.014	0.004	0.008	0.033	0.002	0.011	0.105	0.017	0.006	0.007	0.056	0.012	0.000
**popped**	**QPP2**	0.019	0.029	0.227	0.065	0.013	0.020	0.004	0.006	0.001	0.002	0.021	0.001	0.003	0.021	0.004	0.002	0.003	0.016	0.004	0.000
	**QPP3**	0.029	0.024	0.464	0.136	0.031	0.088	0.007	0.006	0.002	0.002	0.012	0.003	0.004	0.146	0.005	0.003	0.006	0.038	0.006	0.000
	**QPP4**	0.023	0.013	0.218	0.038	0.006	0.026	0.004	0.005	0.001	0.001	0.008	0.001	0.002	0.099	0.002	0.002	0.003	0.012	0.002	0.000
	**QPP5**	0.010	0.005	0.170	0.068	0.006	0.034	0.003	0.002	0.000	0.001	0.005	0.001	0.002	0.015	0.001	0.002	0.001	0.012	0.002	0.000
	**QPP6**	0.017	0.044	0.310	0.106	0.086	0.088	0.007	0.008	0.001	0.001	0.032	0.003	0.004	0.141	0.004	0.004	0.002	0.030	0.005	0.001
	**QPP7**	0.013	0.026	0.482	0.030	0.013	0.035	0.004	0.011	0.001	0.001	0.030	0.001	0.002	0.013	0.001	0.002	0.002	0.011	0.003	0.000
	**QPP8**	0.016	0.017	0.284	0.023	0.009	0.046	0.005	0.006	0.001	0.002	0.010	0.001	0.002	0.064	0.006	0.003	0.003	0.013	0.004	0.000
	**QPP9**	0.012	0.017	0.306	0.150	0.043	0.087	0.004	0.008	0.002	0.003	0.012	0.002	0.005	0.166	0.005	0.004	0.005	0.039	0.005	0.000

	Ala	Arg	Asn	Asp	Gln	Glu	Gly	His	Ile	Leu	Lys	Met	Phe	Pro	Ser	Trp	Thr	Tyr	Val
**non-popped P1/popped P1**	0.93	2.02	4.12	0.87	na	4.79	0.84	1.52	0.97	1.55	1.93	0.97	2.13	0.84	0.53	6.88	1.36	2.44	1.48
**non-popped P2/popped P2**	1.03	1.91	2.63	0.89	na	3.39	1.27	1.34	1.01	1.20	1.87	1.01	1.81	0.26	0.95	1.68	1.46	2.08	1.40
**non-popped P3/popped P3**	1.35	1.32	2.08	1.44	na	1.85	1.06	1.74	1.29	1.41	1.36	1.01	1.81	0.87	0.91	2.72	1.43	2.17	1.31
**non-popped P4/popped P4**	2.33	2.80	4.04	2.50	6.69	5.97	1.22	2.30	na	na	2.28	1.48	2.44	2.25	3.77	4.48	2.94	4.47	1.59
**non-popped P5/popped P5**	1.91	1.72	2.61	1.59	na	2.73	1.17	1.95	2.01	2.01	1.74	2.01	2.40	2.24	1.75	1.90	1.83	2.00	1.69
**non-popped P6/popped P6**	0.88	0.86	1.29	0.96	na	2.05	0.89	1.14	1.22	1.43	1.15	1.27	1.27	0.73	1.22	1.46	1.07	1.31	0.97
**non-popped QPP1/popped QPP1**	1.48	1.15	1.33	1.16	69.13	3.72	1.75	1.74	4.38	4.33	1.78	2.17	2.05	2.18	3.39	1.46	2.34	1.69	3.26
**non-popped QPP2/popped QPP2**	2.35	1.74	2.23	1.49	na	5.42	0.98	2.25	3.10	4.86	2.00	1.45	2.22	0.80	4.63	1.44	2.46	1.90	1.93
**non-popped QPP3/popped QPP3**	1.26	0.88	1.35	1.11	30.13	1.92	1.08	1.12	1.46	1.69	1.00	1.52	1.32	1.33	1.43	1.37	1.49	1.37	1.16
**non-popped QPP4/popped QPP4**	2.78	2.31	3.37	1.03	na	2.25	1.27	3.17	na	2.69	3.19	2.07	3.52	1.12	2.39	2.48	3.11	2.45	1.93
**non-popped QPP5/popped QPP5**	0.69	0.61	1.09	0.90	na	1.00	0.81	0.68	1.01	1.69	0.66	0.91	0.88	0.71	0.88	0.96	0.95	0.80	0.89
**non-popped QPP6/popped QPP6**	1.17	1.32	1.55	1.35	171.42	2.60	1.07	1.57	1.62	1.42	1.36	1.70	1.69	1.07	1.48	1.31	1.22	1.46	1.44
**non-popped QPP7/popped QPP7**	0.82	1.80	2.24	0.34	na	5.04	0.73	3.64	1.77	2.53	4.85	2.03	1.40	0.58	1.35	1.50	1.90	1.07	1.66
**non-popped QPP8/popped QPP8**	2.52	2.32	4.67	0.43	44.31	3.51	0.93	3.11	2.27	3.72	2.97	1.57	2.10	0.82	0.48	1.69	2.33	1.18	1.97
**non-popped QPP9/popped QPP9**	1.45	1.31	2.56	1.47	59.37	3.37	0.89	1.42	1.68	1.84	1.48	1.48	1.60	1.26	2.22	1.64	1.60	1.55	1.61

Supplementary Table S7| Non-popped/popped ratio values of FAA fractions in parental popcorn and colored QPP inbreds.

		Ala	Arg	Asn	Asp	Gln	Glu	Gly	His	Ile	Leu	Lys	Met	Phe	Pro	Ser	Trp	Thr	Tyr	Val
	**QPP1/P1**	3.57	15.40	34.59	8.57	na	8.79	2.20	5.30	1.78	3.95	9.95	0.99	9.09	3.34	2.07	27.21	3.03	13.60	3.03
	**QPP2/P2**	1.55	4.55	13.60	4.71	na	0.92	2.12	1.96	1.02	1.02	4.67	0.82	2.05	1.35	0.62	1.70	1.20	2.45	1.83
	**QPP3/P3**	3.12	5.13	32.25	11.47	na	9.91	2.92	3.53	3.82	2.60	4.38	1.61	5.02	2.22	2.64	2.69	4.10	7.10	3.66
	**QPP4/P4**	4.37	3.27	10.89	14.07	0.00	3.43	1.85	1.87	na	na	1.66	0.99	0.99	10.89	0.90	0.83	1.99	2.23	1.49
**Popped**	**QPP5/P1**	3.17	3.30	10.79	6.39	na	7.92	1.44	2.10	0.98	1.18	3.81	0.98	4.51	1.51	0.44	16.95	1.46	5.96	2.09
	**QPP6/P2**	2.71	9.10	26.87	8.45	na	8.49	3.47	3.98	1.98	1.98	10.38	1.65	4.29	6.76	2.10	3.57	2.10	6.03	2.96
	**QPP7/P4**	8.20	8.39	36.17	33.47	0.00	2.12	2.74	3.60	na	na	3.97	0.99	1.99	2.70	1.35	1.83	2.79	4.68	2.28
	**QPP8/P5**	1.56	2.05	5.61	4.26	na	2.33	2.25	1.12	1.20	1.20	1.46	1.58	1.99	5.46	10.41	1.22	1.33	2.52	1.49
	**QPP9/P6**	1.64	3.38	5.72	6.94	na	4.31	1.52	3.07	2.00	3.00	3.17	1.25	3.51	6.32	1.51	2.44	1.77	5.64	1.87
	**QPP1/P1**	5.69	8.76	11.20	11.43	77.11	6.83	4.60	6.09	8.06	11.06	9.20	2.21	8.75	8.68	13.31	5.77	5.21	9.41	6.67
	**QPP2/P2**	3.55	4.15	11.55	7.83	67.36	1.46	1.63	3.29	3.14	4.14	4.99	1.18	2.52	4.07	3.04	1.46	2.03	2.24	2.52
	**QPP3/P3**	2.92	3.41	20.96	8.87	59.75	10.26	2.98	2.27	4.35	3.12	3.22	2.42	3.65	3.41	4.16	1.36	4.28	4.49	3.25
	**QPP4/P4**	5.21	2.71	9.09	5.78	1.42	1.29	1.92	2.58	1.26	2.73	2.32	1.39	1.44	5.40	0.57	0.46	2.10	1.22	1.80
**Non popped**	**QPP5/P1**	2.37	1.00	2.85	6.62	1.84	1.65	1.39	0.93	1.02	1.29	1.31	0.92	1.87	1.27	0.74	2.35	1.02	1.97	1.25
	**QPP6/P2**	3.10	6.31	15.81	12.74	434.22	6.50	2.93	4.67	3.18	2.34	7.54	2.79	4.02	27.50	3.29	2.80	1.75	4.24	3.04
	**QPP7/P4**	2.90	5.41	20.09	4.50	3.09	1.79	1.63	5.71	1.80	2.57	8.45	1.37	1.14	0.70	0.48	0.61	1.80	1.11	2.37
	**QPP8/P5**	2.06	2.76	10.02	1.16	10.69	3.00	1.79	1.78	1.36	2.22	2.49	1.23	1.75	1.99	2.84	1.09	1.68	1.49	1.74
	**QPP9/P6**	2.69	5.16	11.32	10.66	81.80	7.08	1.51	3.81	2.75	3.86	4.11	1.46	4.41	10.88	2.73	2.75	2.64	6.69	3.10

Supplementary Table S8| QPP/P ratios of FAA in popped and non-popped lines
